# Photodynamic Inactivation of *Staphylococcus aureus* and Biomolecules by Free and Encapsulated Indium(III) Phthalocyanines in PHB Nanoparticles: The Influence of the Position of the Coumarin Group

**DOI:** 10.1021/acs.biomac.4c00862

**Published:** 2025-04-01

**Authors:** Julyana Noval de Souza Ferreira, Barbara Silva Figueiredo, Vannyla Viktória Viana Vasconcelos, Antony Luca Luna Vieira de Abreu, Sheila Souza da Silva Ribeiro, Esra Nur Kaya, Mustafa Bulut, Joselito Nardy Ribeiro, Mahmut Durmuş, André Romero da Silva

**Affiliations:** † Graduate Program in Biochemistry and Pharmacology, Federal University of Espírito Santo, Campus Maruípe, 29047-105 Vitória, Espírito Santo, Brazil; ‡ Federal Institute of Education, Science and Technology of Espírito Santo, Campus Vitória, 29040-780 Vitória, Espírito Santo, Brazil; § Federal Institute of Education, Science and Technology of Espírito Santo, Campus Aracruz, 29192-733 Aracruz, Espírito Santo, Brazil; ∥ Faculty of Art and Science, Department of Chemistry, Marmara University, 34722 Kadıköy, İstanbul, Turkey; ⊥ Department of Chemistry, Gebze Technical University, 41400 Gebze, Kocaeli, Turkey; # Health Science Center, Federal University of Espírito Santo, 29043-910 Vitória, Espírito Santo, Brazil

## Abstract

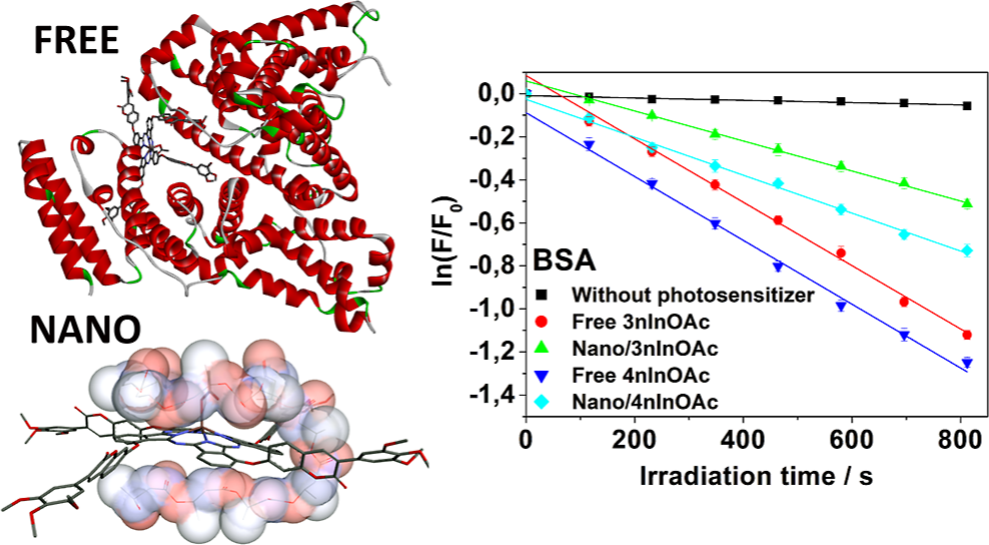

Antimicrobial photodynamic therapy (APDT) is a promising alternative to inactivating resistant microorganisms. Metallic phthalocyanines (Pc) substituted with coumarin groups exhibit favorable photophysical properties for APDT; however, their hydrophobicity limits administration. This study investigates indium(III) Pc substituted with 7-oxy-3-(3′,4′,5′-trimethoxyphenyl)coumarin at nonperipheral (**3nInOAc**) and peripheral (**4nInOAc**) positions, both in their free form and encapsulated in polyhydroxybutyrate nanoparticles, for the photodynamic inactivation of methicillin-resistant *Staphylococcus aureus* (MRSA) and methicillin-susceptible *Staphylococcus aureus* (MSSA) bacteria. The photodynamic activity was also assessed through the photooxidation of tryptophan and bovine serum albumin. Theoretical calculations and molecular docking were performed to corroborate the experimental results, investigating the influence of molecular structure on the photodynamic and antimicrobial performance of Pc-loaded nanoparticles as well as their nanoparticulate properties. Overall, both free and encapsulated Pc were capable of photooxidizing biomolecules and exhibited moderate antimicrobial activity, with **4nInOAc** demonstrating superior efficacy, achieving an average reduction of 2 logs (99%) in MSSA and MRSA colonies.

## 1 Introduction

Antimicrobial resistance (AMR) is declared by the World Health Organization (WHO) as one of the main threats to public health currently facing humanity.^1^ It was estimated that 5 million people died in 2019 due to diseases caused by drug-resistant microorganisms and that 10 million deaths could occur per year by 2050.^2,3^ Especially with the SARS-CoV-2 coronavirus pandemic, the threat of AMR has gotten worse due to the increase in the use of antibiotics.^4,5^ Without effective antimicrobials, the treatment of infections is becoming more and more challenging, increasing mortality and the risk of surgical procedures.^6−8^

In February 2017, WHO published a list of priority antibiotic-resistant pathogens that pose the greatest epidemiological threat and that should be considered a priority in the research for treatment alternatives.^9^ One of these pathogens highlighted as a high priority is *Staphylococcus aureus*, a Gram-positive bacterium that cause infections both in the community and in healthcare facilities, and developed resistance to several commonly used antibiotics.^10^ Infections caused by methicillin-resistant *Staphylococcus aureus* (MRSA) have a probability of mortality that is 64% higher than that of infections caused by the drug-sensitive strain.^1,11^

AMR is a complex problem that requires new approaches that are effective in the treatment and control of these resistant microorganisms. An alternative that has been standing out and showing satisfactory results is antimicrobial photodynamic therapy (APDT), also known as photodynamic inactivation of microorganisms, which aims to combat localized infections without use systemic drugs to eliminate the possibility of accelerating the emergence of infections that are resistant to antibiotics.^12−15^ APDT involves the action of a photosensitizer (PS), which is activated by a light irradiation at a specific wavelength and oxygen molecules to generate cytotoxic reactive oxygen species (ROS), capable to oxidize biomolecules and cause the microorganisms death.^15−17^ Phthalocyanine (Pc) derivatives have a great potential as PSs due to their intense light absorption in the “therapeutic window” (600–800 nm) and because these compounds have a good capacity to generate singlet oxygen, an important factor for the effectiveness of the photodynamic action.^18−20^ The presence of heavy atoms in the structure of the molecule or the substituent groups in the nonperipheral and peripheral positions on the Pc macrocycle can still favor the generation of the singlet oxygen.^21−24^ The tetrakis indium(III) Pc substituted with 7-oxy-3-(3′,4′,5′-trimethoxyphenyl)coumarin groups on the peripheral and nonperipheral positions (**4nInOAc** and **3nInOAc**, respectively) showed high singlet oxygen quantum yields (0.78 and 0.91, respectively) and low photodegradation quantum yield (2.37 × 10^–4^ and 0.60 × 10^–4^, respectively), suggesting that these hydrophobic compounds have excellent ability transferring energy to molecular oxygen and consequently, to generate the ROS.^23^ The trimethoxyphenyl coumarin groups have also decreased the photodegradation quantum yield values, increasing the stability of the coumarin substituted Pc complexes to generate singlet oxygen and favor a better result for photodynamic therapy.^23,25^ Pc substituted with *m*-methoxyphenyl coumarin groups showed the same photochemical results.^26^ It is known that larger indium metal in the cavity of the Pc favors the intersystem crossing between singlet state and triplet state, resulting in higher values of singlet oxygen quantum yield (heavy atom effect) and a lower value of fluorescence quantum yield.^26,27^

However, the most of the molecules used as PSs, including Pc, are hydrophobic, which favors their aggregation in aqueous media,^28,29^ hampering their systemic administration and affecting their bioavailability. PSs with a planar aromatic π system and rigid structure have a strong intrinsic tendency to form stable aggregates depending on the peripheral groups bound in the molecule. The aggregation can influence on the photodynamic therapy activities of the PSs.^24,28,30^ Results have showed that the encapsulation of the hydrophobic PS into the polymeric nanoparticle of poly(3-hydroxybutyrate) (PHB) improved the solubility and the photodynamic efficacy of the compounds.^21,31^ Despite advances, the use of PHB polymeric nanoparticles containing Pc for application in APDT has not been much explored. PHB is a biocompatible and biodegradable polymer, approved by the Food and Drug Administration (FDA, USA) that have been used to synthesize drug delivery system.^32−34^

It has been a challenge to scale up of the nanoparticle formulation because of the different preparation method and the influence of variables, and their synergistic and antagonistic effects, on the properties of nanoparticles.^35,36^ The same parameter may cause distinct outcomes even for similar nanoparticulate formulations, therefore each nanoparticulate formulation should be treated singularly.^36^

Biomolecules have been used as a model to evaluate the photodynamic efficiency of a PS.^24,37^ In particular, tryptophan (Trp) has stood out as an amino acid highly susceptible to oxidation in physiological pH, present in important proteins that constitute the subcellular targets of photodynamic therapy.^38^ Similarly, a protein that has been used as a model in several photodynamic studies is bovine serum albumin (BSA),^37,39^ which also contains Trp.

Therefore, this work aimed to evaluate the influence of the tetrakis indium(III) Pc bearing [7-oxy-3-(3′,4′,5′-trimethoxyphenyl)coumarin] groups on the peripheral or nonperipheral positions of the Pc macrocycle (**4nInOAc** and **3nInOAc**, respectively). The PS mass and the stirring rate on the size, the recovery efficiency of nanoparticles, and the entrapment efficacy of the PHB nanoparticles loaded with the **3nInOAc** or **4nInOAc** prepared following a 2^3^ factorial design. The photodynamic activity of each free and encapsulated PS was evaluated by the photooxidation of biomolecules Trp and BSA. Theoretical calculations and molecular docking studies were performed to explain the entrapment efficiency (EE) of PSs in PHB nanoparticles and the effectiveness in photooxidizing biomolecules. Finally, we assayed the in vitro photodynamic antimicrobial efficiency of **3nInOAc** and **4nInOAc** free and encapsulated in PHB nanoparticles against MRSA and methicillin-susceptible *S. aureus* (MSSA).

## 2 Experimental Section

### 2.1 Reagents

**Figure 1:**
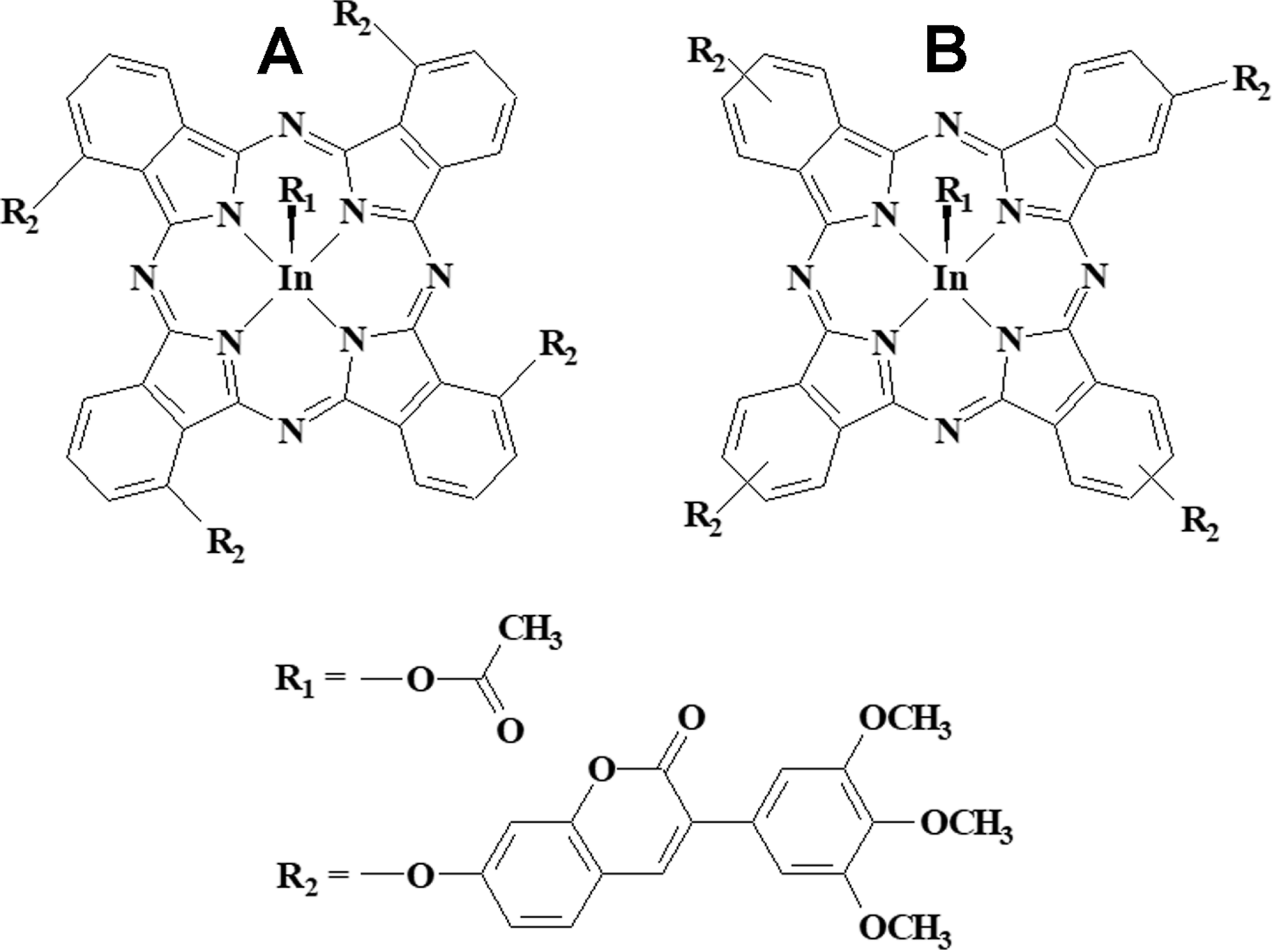
Molecular structures of (A) 2(3),9(10),16(17),23(24)-Tetrakis[7-oxo-3-(3′,4′,5′- trimethoxyphenyl)coumarin] phthalocyaninato indium(III) acetate (**3nInOAc**), (B) 1(4),8(11),15(18),22(25)-Tetrakis[7-oxo-3-(3′,4′,5′-trimethoxyphenyl)coumarin] phthalocyaninato indium(III) acetate (**4nInOAc**).

2(3),9(10),16(17),23(24)-Tetrakis[7-oxo-3-(3′,4′,5′-trimethoxyphenyl) coumarin] phthalocyaninato indium(III) acetate (**3nInOAc**) and 1(4),8(11),15(18),22(25)-Tetrakis[7-oxo-3-(3′,4′,5′-trimethoxyphenyl) coumarin] phthalocyaninato indium(III) acetate (**4nInOAc**) (Figure 1) used in this work as PSs were synthesized and characterized by Kaya et al. according to their previous work.^23^

The methyl-2-pyrrolidone, poly(vinyl alcohol) (PVA) (Mw = 13,000–23 000 Da, 87–89% hydrolyzed), BSA, and l-tryptophan (Trp) were purchased from Sigma-Aldrich (USA). Dimethyl sulfoxide (DMSO), Tween 20, and chloroform were obtained from Vetec Química Fina Ltd (Brazil). Trypticase soy agar (TSA) was obtained from Oxoid Ltd. (England). Trypticase soy broth (TSB) and bacteriological peptone were obtained from Kasvi (Brazil). The water used throughout the experiments was first bidistilled and then deionized (Millipore, USA). All other chemicals used were of analytical grade and were used without further purification.

### 2.2 Theoretical Calculations for Optimization of Pc Structures and Molecular Docking

To determine the physicochemical properties of the optimized Pc molecules, the three-dimensional structure of **3nInOAc** and **4nInOAc** molecules were designed in Avogadro 1.2.0 software, using the universal force field (UFF) for geometric optimization.^40^ Then, the optimization of these structures was performed using the semiempirical method parametric method number 7 (PM7) in MOPAC 2016 software (MOPAC 2016TM,^41^ Stewart Computational Chemistry, USA), obtaining the values of surface area and volume of the molecules. The hydration energy was also obtained by subtracting the energy of formation calculated by the COSMO method in the presence of water (solvent), considering its relative permittivity and the van der Waals radius, from the energy calculated by the PM7 method in the absence of water.^41^ Log *P* and polarizability values were obtained using MarvinSketch 20.20 Program with the molecules optimized by MOPAC software.

To perform the molecular docking, the PHB monomer was designed and optimized in Avogadro 1.2.0 software using a variant of the Merck molecular force field (MMFF94s). Then, using the PyMol Molecular Graphics System Program (Version 2.4.1 Schrödinger, LLC Pymol), its replication to the decamer was performed. Furthermore, the decamer random conformational analysis was performed by Avogadro 1.2.0 using the MMFF94s force field, following optimization by the PM7 method using MOPAC.

After the optimization step, the addition of charges to the optimized structures of the Pc molecules and the polymer decamer was performed using the USCF Chimera 1.14 program (UCSF Resource for Biocomputing, Visualization, and Informatics).^42^

Molecular docking simulations were performed by using AutoDock Vina software. The PHB molecule was labeled as a ligand, and the Pc as receptors, to which polar hydrogens were added. Then, to calculate the prediction of receptor–ligand interactions, Pc was surrounded by a cubic grid box with micro boxes separated by 0.1 nm (small grid box). Thus, all the parameters necessary to perform the molecular docking calculations were added to a text file, as described below, for the **3nInOAc** and **4nInOAc** Pc, respectively.
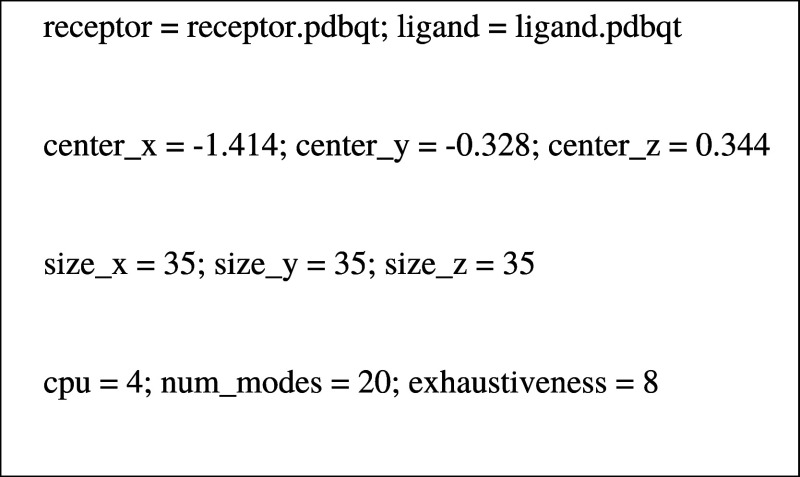

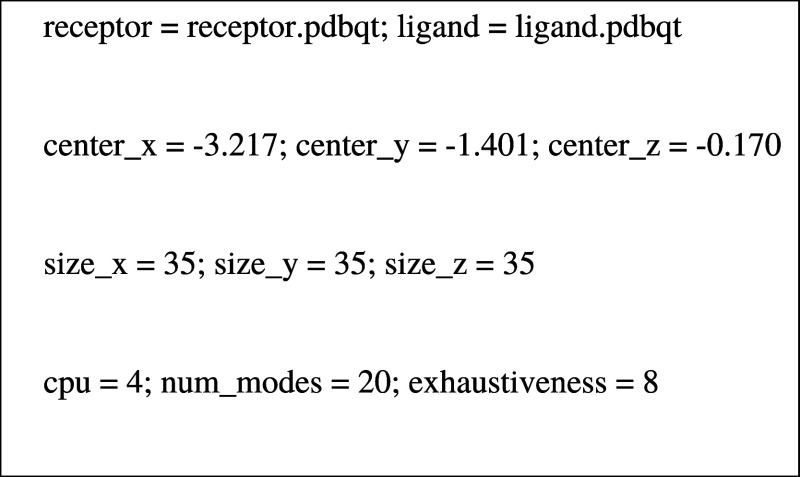


The docking simulations were performed, indicating the interaction energies between the receptor and the ligand. Ten docking simulations were performed for each Pc complex, and the best result was chosen for each complex.^43^

To perform the molecular docking calculations of the protein binding with Pc, the three-dimensional structure of the BSA was obtained from the Protein Data Bank (https://www.rcsb.org; code 4OR0), then visualized and edited with the PyMol 2.4.1 program, removing the naproxen ligand. Previously, Pcs were optimized by semiempirical methods using AutoDock Vina software. Pcs were labeled as ligands, and BSA was treated as a receptor, to which polar hydrogens were added. Then, to calculate the prediction of receptor–ligand interactions, the BSA molecule was surrounded by a cubic, small grid box. The settings given below were used for docking calculations. Ten simulations were performed for each complex, and the best result was chosen.
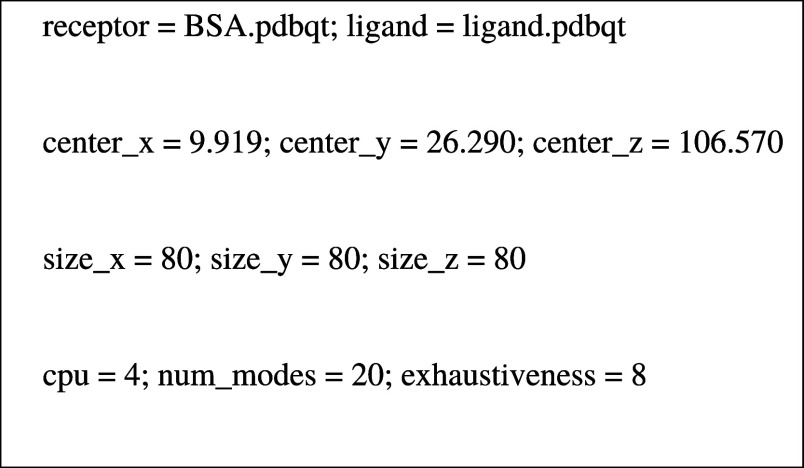


### 2.3 2^3^ Factorial Design Applied to the Preparation of PHB Nanoparticles Containing **3nInOAc** or **4nInOAc**

The influence of three parameters involved in the preparation of the nanoparticles was evaluated on the size, which was estimated by the integrate area of absorbance spectra (IAAS), the EE, and the efficacy of nanoparticles recovery (ER). The analyses were carried out using a 2^3^ factorial design consisting of two levels and center point runs. The factors, levels, and the matrix of 2^3^ factorial design are shown in Tables 1 and 2, respectively. The factors and the values of the levels were chosen on previous results obtained by our research group.^21,36^ All experiments were performed in duplicate, and statistical analysis of experimental data was performed by Statistica 6.0 software using analysis of variance (ANOVA). The results were expressed by using the Pareto chart.

**Table 1:** Factors and Levels Investigated in the 2^3^ Factorial Design

factor		levels		
		–	0	+
A	position of the substituent group [7-oxy-3-(3,4,5-trimethoxyphenyl)coumarin] on the indium(III) Pc	nonperipheral		peripheral
		**3nInOAc**		**4nInOAc**
B	PS mass/mg	0.14	0.21	0.28
C	stirring rate/rpm	10,000	6000	22,000

**Table 2:** Matrix of 2^3^ Factorial Design^a^

experiments n°	factor		
	A	B	C
1	–	–	–
2	+	–	–
3	–	+	–
4	+	+	–
5	–	–	+
6	+	–	+
7	–	+	+
8	+	+	+
9	–	0	0
10	+	0	0

a The plus sign indicates the higher level, the minus sign indicates the lower, and zero indicates the center point.

### 2.4 Preparation of PHB Nanoparticles Containing **3nInOAc** or **4nInOAc**

The polymeric nanoparticles were prepared by the emulsion/evaporation method at a room temperature of 21 °C.^44,45^ First, a solution of PHB was prepared using chloroform as the solvent (12 g/L), followed by the preparation of a Pc solution, dissolving the mass of PS (Tables 1 and 2) in *N*-methyl-2-pyrrolidone (4 mL). An organic phase was prepared by adding a volume of PHB solution (8 mL) into the Pc solution (4 mL).

The organic phase was slowly added to the aqueous phase (70 mL) containing a PVA solution (2% m/v). This mixture was homogenized for 7 min at a stirring rate of 10,000, 16,000, or 22,000 rpm (UltraTurrax T25, IKA USA), according to the factorial design (Tables 1 and 2). The resulting emulsion was maintained under reduced pressure in a rotary evaporator for 1.5 h and then under magnetic stirring for 24 h to evaporate the organic solvent. Afterward, the particles were recovered by centrifugation at 60,000*g* for 20 min at 19 °C (Beckman Avanti J30-i, Beckman Instruments, USA) and washed 4 times with water to remove excess PVA and nonincorporated Pc. Then, the final particle suspension was lyophilized for 48 h at 28 μm Hg and −45 °C (LioBras, LIOTOP L101, Brazil), using glucose (0.4% m/v) as a cryoprotectant.

### 2.5 Size and Morphology of the Nanoparticles

An estimation of the particle size was performed by measurement of the IAAS of the suspensions from 400 to 600 nm. In this spectral range, the light is scattered only by the particles.^46−48^ All absorbance measurements were performed using water as a blank. The IAAS was calculated using OriginPro 2016 software (OriginLab Corp., USA), and the value obtained was considered proportional to the colloidal suspension turbidity.

The average size as well as the size distribution of the nanoparticles were evaluated by dynamic light scattering (NPA152 Zetatrac, Microtrac, USA). Zeta potential was also evaluated for the formulations that presented the best results using the Litesizer 500 equipment (Anton Paar, AT), as well as the morphology of the particles by scanning electron microscopy (SEM, JSM-6610 LV, JEOL, Tokyo, Japan). For this morphological analysis, a droplet of the nanoparticle suspension was placed directly on a metallic stub, air-dried, and then coated with a thin layer of gold. The samples were observed under a microscope without any further treatment.

### 2.6 Entrapment Efficiency and Nanoparticle Recovery

A previously known amount of the lyophilized samples was dissolved in *N*-methyl-2-pyrrolidone (10 mL) for quantification of **3nInOAc** and **4nInOAc** by UV–vis absorption spectroscopy (Agilent Cary 50 Conc, USA). The measurement was performed at maximum absorbance wavelength for each compound (705 nm for **3nInOAc** and 690 nm for **4nInOAc**), and an analytical calibration curve was obtained from ten different concentrations of **3nInOAc**/**4nInOAc** for the quantification. The EE of each Pc in the polymeric particle was calculated using eqs 1–3.^49^
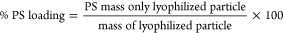
1
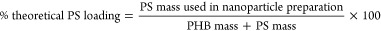
2
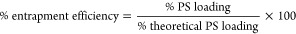
3

The recovery efficacy was calculated by considering the mass of lyophilized particles as well as the mass of PHB and PS used in the nanoparticle preparation, as shown in eq 4.^49^
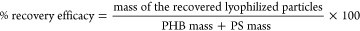
4

### 2.7 BSA and Trp Photooxidation by Free and Encapsulated **3nInOAc** and **4nInOAc**

500 μL of a BSA solution (320 μmol/L) prepared in phosphate-buffered saline (PBS) solution (pH 7.4), 60 μL of free **3nInOAc** or **4nInOAc** solution dissolved in DMSO (500 μmol/L), 15 μL of Tween 20 (5% m/v), and 1425 μL of the PBS solution were added in a vial, so that the final PS concentration was 15 μmol/L. An aliquot of 1.7 mL of this solution was transferred to a microtube and irradiated using a laser diode, INOVA 665 nm from Laserline (Brazil), with a power of 104 mW, irradiance of 14.7 mW/mm^2^, and seven light doses of irradiation of 1 J/cm^2^ for 2 min each. BSA emission fluorescence was monitored using a Cary Eclipse Fluorescence Spectrophotometer (Agilent Technologies, Santa Clara, USA) before the first irradiation (*F*_0_) and after each light dose (*F*). BSA was excited at 250 nm, and the fluorescence intensity was monitored from 260 to 450 nm. The integrated area of the fluorescence spectrum, before and after irradiation, was calculated using OriginPro 2016 software, and a plot of natural logarithm ln (*F*/*F*_0_) versus time was obtained to determine the first-order rate constant for the BSA photooxidation.

For the encapsulated Pc, the final concentration of each PS was the same as that used in the experiments carried out with the free Pc (15 μmol/L). 1000 μL of particles dispersion in PBS (30 μmol/L), 500 μL of a solution of BSA (320 μmol/L), 15 μL Tween 20 (5% m/v), and 485 μL of PBS solution were added in a vial. After the same irradiation procedure was performed, the dispersion was centrifuged for 5 min at 15,000*g* (CT-15000, CIENTEC, Brazil) and then the fluorescence of the supernatant was measured using the same parameters used for the photooxidation assays with the free Pc, to monitor the BSA spectrum profile.

The Trp photooxidation was performed in the same way as the BSA photooxidation for the free and encapsulated Pc, except that a more concentrated Trp stock solution was used (2500 μmol/L). Then, to maintain the same final concentrations used for BSA, 64 μL of this Trp solution was transferred to a vial, keeping the same volumes as the Pc and Tween 20 solutions. The Trp molecule was excited at a wavelength of 250 nm, and the fluorescence intensity was measured from 260 to 450 nm. *F* and *F*_0_ values were monitored by fluorescence measurements, and the plot of ln (*F*/*F*_0_) versus irradiation time was obtained to determine the photooxidation rate constant of Trp.

All procedures were performed in triplicate. To evaluate the ability of the laser to cause the photooxidation, irradiations were also performed in BSA and Trp solutions in the absence of the PSs (light control). Statistical analyses were carried out using ANOVA followed by the Tukey’s Multiple Comparison Test with a significance level of *p* < 0.05.

### 2.8 In Vitro APDT

#### 2.8.1 Bacterial Strains and Culture Conditions

MSSA ATCC 29213 and MRSA ATCC 43300 were used for this study. These strains were maintained on TSA at 4 °C, and prior to each APDT assay, a colony was transferred to 30 mL of TSB to grow at 37 °C until the stationary phase achieves 10^8^ CFU/mL. Afterward, a 1/10 dilution in bacteriological peptone was prepared and used for the photodynamic inactivation assays (cellular density: 10^7^ CFU/mL).

#### 2.8.2 Photosensitizers

For the assays with free Pcs, stock solutions were prepared at 100 μmol/L in DMSO/PBS (1:9) (pH 7.4), with a final concentration of DMSO of 1% (v/v). The DMSO present in the final dilutions did not change the bacterial viability. For the assays with the Pc encapsulated in polymeric nanoparticles, formulations were dispersed in PBS (pH 7.4) at the same concentration as the free compound solutions (100 μmol/L) but in the absence of DMSO.

#### 2.8.3 Photodynamic Inactivation Assays

An aliquot of 500 μL of the microorganism cell suspension was added to a microtube with 500 μL of the free or encapsulated PS solution, reaching a final concentration of 50 μmol/L PS and a cell density of 10^7^ CFU/mL. This solution was homogenized using a Vortex shaker (XH-2800, Warmnest). Afterward, 60 min of dark incubation was performed under stirring (200 rpm) at 37 °C (Incubator Shaker, Marq Laboratories) to promote the binding of the PS to the microorganisms. Then, this suspension was irradiated using a laser diode, INOVA 665 nm from Laserline (Brazil) with a power of 104 mW, irradiance of 14.7 mW/mm^2^, and a light dose of irradiation for 60 min to deliver 29.5 J/cm^2^.

After the irradiation period, aliquots of 100 μL were removed from the irradiated samples and serially diluted 10-fold to give dilutions of 10^–1^ to 10^–5^ times the original concentrations. An aliquot of 100 μL of each dilution was cultured on TSA plate in duplicate. Then, the number of colonies formed after 24 h incubation at 37 °C was counted. Data (CFU/mL) were log-transformed [Log_10_ (CFU/mL)]. Three independent assays for each condition were performed, and the data were expressed as the average of all values obtained.

Control groups included bacteria that were not treated with PSs or light [growth control (GC)], bacteria treated with light in the absence of PSs [light control (LC)], and bacteria treated with PSs in the absence of light (dark control, DC_PS3_ and DC_PS4_ for **3nInOAc** and **4nInOAc**, respectively). Photodynamic assays included the free and encapsulated PSs (PS_3free_, PS_4free_, PS_3encap_, and PS_4encap_), and the results were compared with the control cultures (GC) to evaluate the photodynamic action of the PSs in the inactivation of bacteria. Statistical analyses were carried out using ANOVA followed by the Tukey’s multiple comparison test with a significance level of *p* < 0.05.

## 3 Results and Discussion

### 3.1 Characterization and Theoretical Calculations for Optimization of Pc Structures and Molecular Docking

**Figure 2:**
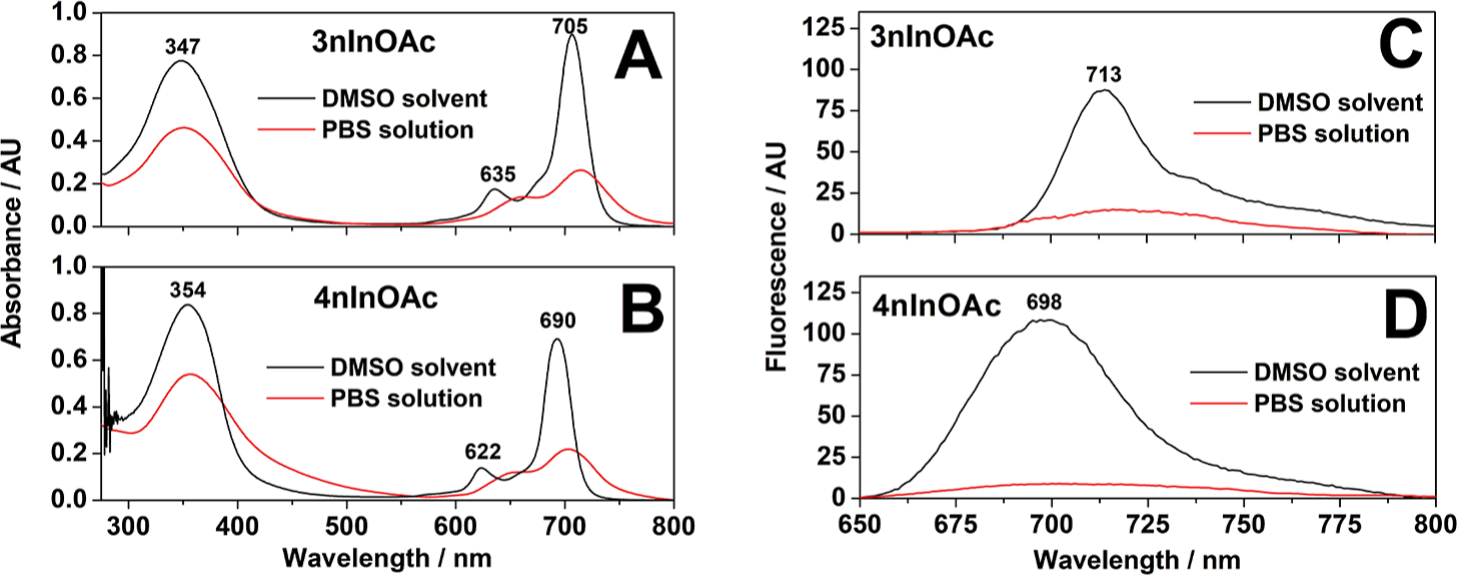
Absorbance and fluorescence spectrum profile of the free **3nInOAc** (A,C, respectively) and **4nInOAc** (B,D, respectively) in the concentration of 7.5 μmol/L in DMSO and pH 7.4 PBS solution, containing 3% (v/v) of the organic solvent and 5% (m/v) of the Tween 20.

The absorbance spectrum profiles of free **3nInOAc** (Figure 2A) and **4nInOAc** (Figure 2B) in DMSO showed the characteristic bands of Pc compounds at 300–400 nm (Soret band) and 600–750 nm (Q-band). In addition, the **3nInOAc** (Figure 2C) and **4nInOAc** (Figure 2D) presented a fluorescence spectrum profile with typical bands of Pc from 650 to 800 nm. However, the absorbance and fluorescence intensities were decreased when the compounds were dissolved in aqueous PBS solution due to molecular aggregation (Figure 2).^24,29^

**Figure 3:**
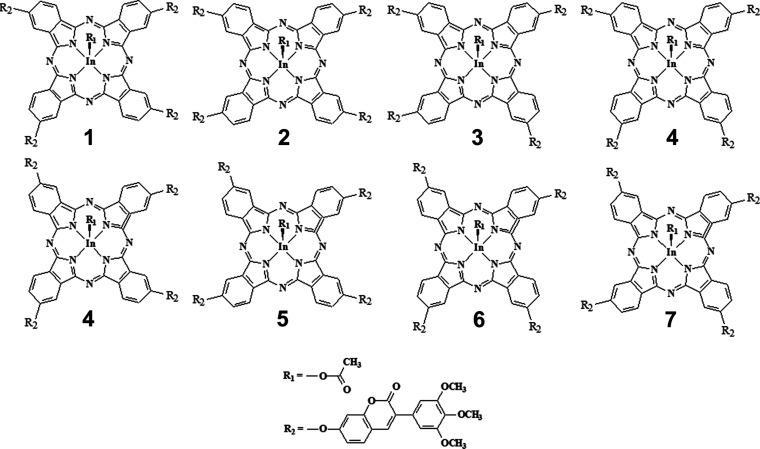
Structural isomers of **4nInOAc** PS.

The Pc **4nInOAc** is formed by a mixture of eight structural isomers (Figure 3) according to its molecular structure (Figure 1). Therefore, the physicochemical properties (Table 3) and the molecular docking of Pc with the polymer (PHB) and with the protein (BSA) were calculated for each isomer of this compound (Table 4).

**Table 3:** Physicochemical Properties of the **4nInOAc** Structural Isomers, Calculated by Semiempirical Methods

molecules		properties				
		asurface area (Å^2^)	avolume (Å^3^)	bLog *P*	bpolarizability	ahydration energy (kcal/mol)
**4nInOAc**	isomer 1	1681.77	2007.58	11.63	198.72	91.12
	isomer 2	1686.31	2012.38	13.41	202.12	79.68
	isomer 3	1682.96	2012.07	11.63	198.72	90.63
	isomer 4	1687.98	2019.37	11.63	198.72	92.11
	isomer 5	1687.30	2012.72	12.56	199.85	96.80
	isomer 6	1687.30	2012.72	12.56	199.85	96.80
	isomer 7	1687.30	2012.72	12.56	199.85	96.80
	isomer 8	1687.98	2019.37	11.63	198.72	92.11

a Values were calculated from MOPAC 2016 software.

b Values estimated by MarvinSketch 20.20 software.

**Table 4:** Interaction Energies of **4nInOAc** Structural (Pc) with Polymer (PHB) and Albumin (BSA), Calculated by Molecular Docking

molecules		properties	
		Δ*G* (Pc-polymer) (kcal/mol)	Δ*G* (Pc-BSA) (kcal/mol)
**4nInOAc**	isomer 1	–4.2	–12.1
	isomer 2	–4.1	–12.5
	isomer 3	–4.6	–12.6
	isomer 4	–4.2	–12.5
	isomer 5	–4.5	–12.1
	isomer 6	–4.3	–12.3
	isomer 7	–4.4	–12.3
	isomer 8	–4.1	–12.3

The surface area values changed from 1681.77 Å^2^ (isomer 1) to 1687.98 Å^2^ (isomers 4 and 8), while the molecular volume ranged from 2007.50 Å^3^ (isomer 1) to 2019.37 Å^3^ (isomer 4 and 8) (Table 3). Results showed that the isomers with the lowest value of the surface area had the smallest molecular volume, while the isomers with the largest surface area showed the largest volume. Log *P* value changed from 11.63 (isomers 1, 3, 4, and 8) to 13.41 (isomers 2), while the polarizability changed from 198.72 (isomers 1, 3, 4, and 8) to 202.12 (isomer 2) (Table 3). These results disclosed that the isomers with the smallest Log *P* value showed the smallest polarizability, while the isomer with the highest Log *P* value presented the highest polarizability. Hydration energy changed from 79.68 (isomer 2) to 96.80 kcal/mol (isomers 5, 6, and 7) (Table 3). These results showed that the more hydrophobic isomer (isomer 2) presented the smallest value of the hydration energy, suggesting this isomer 2 could be less solvated by water molecules if compared with the isomers 5, 6, and 7. The interaction energy of each **4nInOAc** isomer with PHB polymer changed from −4.1 (isomers 2 and 8) to −4.6 kcal/mol (isomer 3), while the interaction energy of each isomer with BSA changed from −12.1 (isomers 1 and 5) to −12.6 kcal/mol (isomer 3) (Table 4). Results suggest that isomer 3 could have a greater affinity with PHB and BSA molecules if compared with other isomers. Considering the small difference between the smallest and highest values of surface area, volume, polarizability, and the **4nInOAc**-BSA interaction of isomers (0.37%, 0.58%, 1.68%, and 3.97%, respectively), and that the concentration of each isomer is not known, we decided to estimate the average value of all properties to compare with the **3nInOAc** properties. However, the difference between the lowest and highest values of Log *P*, hydration energy, and the **4nInOAc**-polymer was 13.3%, 17.7%, and 10.87%, respectively, suggesting that the hydrophobicity of the isomers is not similar, allowing that each isomer may have a different behavior in the photooxidation reactions performed in the aqueous phase.

As the PSs differ only in the nonperipheral and peripheral position of the substituent on the indium(III) Pc, it is plausible that there is small difference between the physicochemical properties of the **3nInOAc** and **4nInOAc** (Table 5). However, results suggest that **4nInOAc** is more hydrophilic than **3nInOAc**, according to the lower values of Log *P* and polarizability and the higher value of hydration energy. The effect of these differences between both Pc on the nanoparticle properties and photodynamic efficiency will be discussed later.

**Table 5:** Physicochemical Properties of the **3nInOAc** and **4nInOAc**, Calculated by Semiempirical Methods and Molecular Docking

properties	molecule	
	**3nInOAc**	d**4nInOAc**
asurface area/Å^2^	1661.04	1686.11
avolume/Å^3^	2042.14	2013.62
bLog *P*	14.41	12.20
bpolarizability	202.14	199.57
ahydration energy/kcal/mol	32.80	92.01
cΔ*G* (Pc-polymer)/kcal/mol	–4.7	–4.3
cΔ*G* (Pc-BSA)/kcal/mol	–11.1	–12.3

a Values were calculated from MOPAC 2016 software.

b Values estimated by MarvinSketch 20.20 software.

c Values were calculated by molecular docking from Autodocking Vina.

d Average values obtained for **4nInOAc** isomers.

### 3.2 Influence of Factors A, B, and C on the IAAS of the **3nInOAc** or **4nInOAc**-Loaded Nanoparticles

The nanoparticles size was evaluated by calculating the integrated area of absorbance spectra (IAAS) (Table 6) in the spectrum range between 400 and 600 nm, which the Pc does not absorb electromagnetic energy. The scattering of electromagnetic radiation by a particle is directly proportional to the square of nanoparticle volume (*I*_sct_ ∝ *V*^2^);^48^ thus, a suspension of nanoparticles with a small diameter presents an absorbance spectral profile with smaller integrated area due to lower light scattering. Therefore, the smaller the size of the nanoparticles, the lower the turbidity of nanoparticulate suspension, and consequently, the lower the IAAS value, allowing the particle size of the formulations to be compared using the IAAS values.

**Table 6:** Integrated Area of the Absorbance Spectrum (IAAS), EE, and Recovery Efficacy (RE) of the **3nInAOc** or **4nInAOc**-Loaded PHB Nanoparticles from the 2^3^ Factorial Design (Table 2)

exp	A	B	C	IAAS	%EE	%RE
1	–	–	–	510 ± 17	86 ± 2	87 ± 4
2	+	–	–	532 ± 10	88 ± 3	79 ± 13
3	–	+	–	529 ± 4	62 ± 4	62 ± 4
4	+	+	–	523 ± 8	60 ± 4	53 ± 9
5	–	–	+	194 ± 1	69 ± 3	86 ± 4
6	+	–	+	201 ± 17	57 ± 1	50 ± 5
7	–	+	+	340 ± 160	51 ± 3	48 ± 19
8	+	+	+	175 ± 18	46 ± 4	39 ± 1
9	–	0	0	438 ± 25	68 ± 8	51 ± 4
10	+	0	0	405 ± 15	66 ± 3	49 ± 2

**Figure 4:**
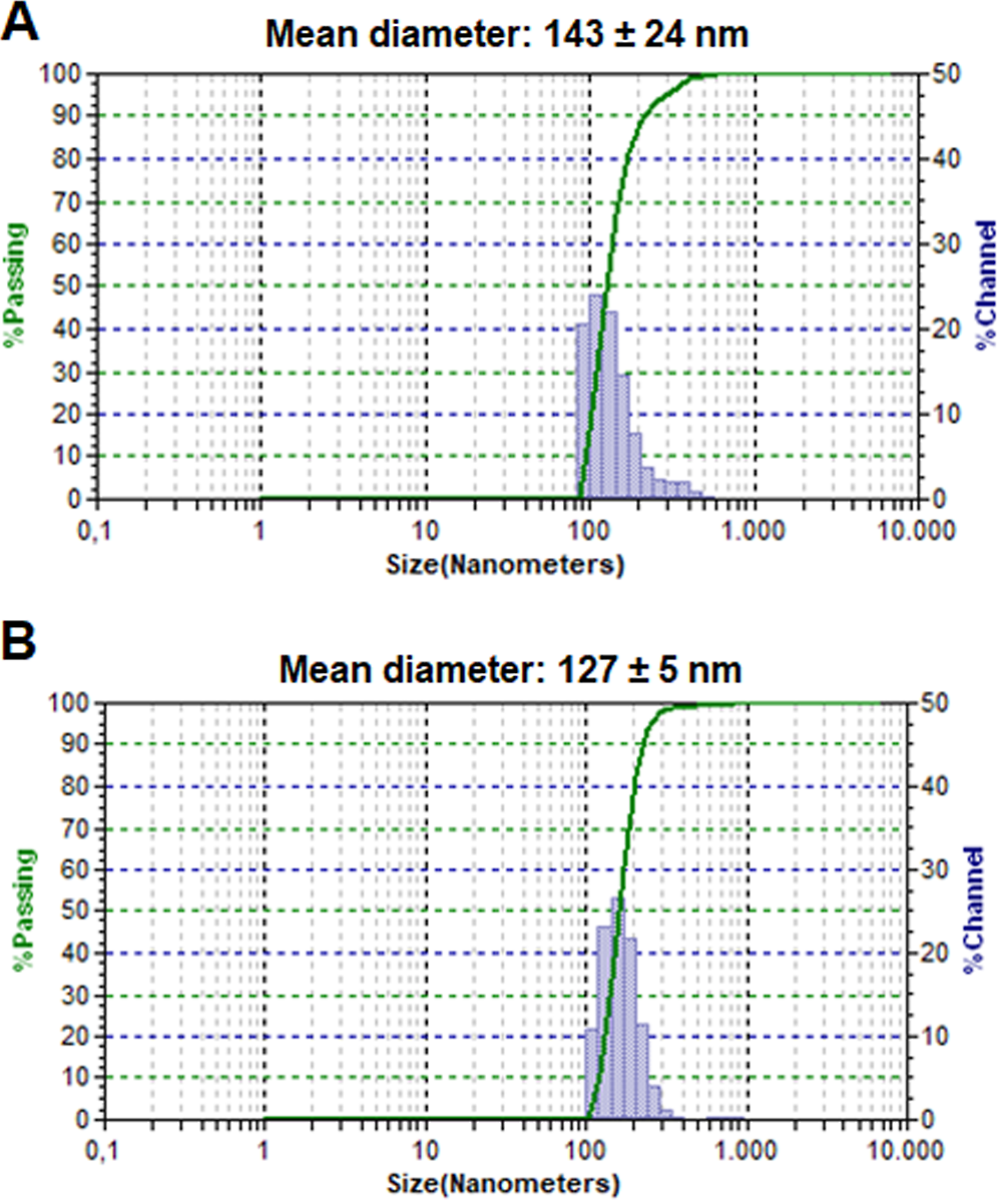
Size distribution of PHB nanoparticles prepared according to experiments 5 and 8 of the 2^3^ factorial design loaded with (A) **3nInOAc** and (B) **4nInOAc**, respectively.

IAAS values ranged from 194 ± 1 (experiment 5) to 529 ± 4 (experiment 3) (Table 6) for the nanoparticles loaded with **3nInOAc**, indicating that the levels used in experiment 5 were the most suitable for the preparation of polymeric nanoparticles of smaller sizes, while the conditions used in experiment 3 favored obtaining larger particles. Dynamic light scattering analysis revealed that the experiment 5 formulation was characterized by particles with average mean diameters of 143 ± 24 nm, with 88.8% of the particles having diameters between 86 and 204 nm (Figure 4A). However, IAAS values ranged from 175 ± 18 (experiment 8) to 532 ± 10 (experiment 2) (Table 6) for the particles loaded with **4nInOAc**, disclosing that the parameters applied in experiment 8 favored the obtaining of smaller particles. This formulation was characterized by particles with an average mean diameter of 127 ± 5 nm, with 82.1% of the particles with diameters between 86 and 204 nm (Figure 4B). It is important to know that IUPAC and ISO define nanoparticles as materials characterized with size from 1 to 100 nm.^50,51^ However, nanoparticles were defined by Encyclopedia of Pharmaceutical Technology as a solid colloidal particle ranging in size from 1 to 1000 nm (1 μm).^52−54^ Researchers also have defined nanoparticles and submicron particles as materials with nanometric sizes (from 1 to 100 nm, and from 100 to 1000 nm, respectively).^55^ Considering that this work synthesized and evaluated polymeric particles to be used in photodynamic therapy, we decided to maintain the nanoparticle definition of the Encyclopedia of Pharmaceutical Technology for particles loaded with **3nInOAc** and **4nInOAc**.

**Figure 5:**
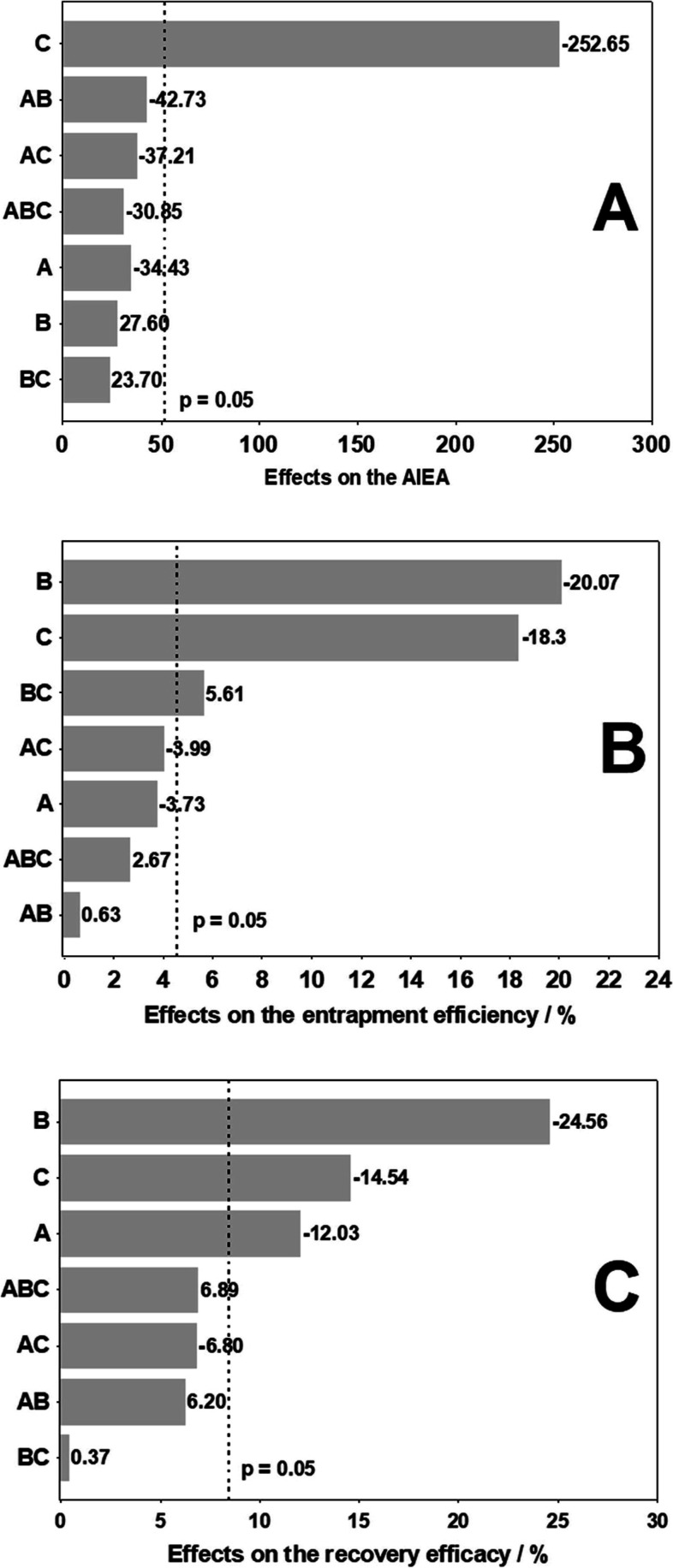
Main and combinatory effects of three parameters [Factor A = peripheral or nonperipheral position of the substituent group [7-oxy-3-(3′,4′,5′-trimethoxyphenyl)coumarin] on the indium(III) Pc; Factor B = PS mass; Factor C = Stirring rate] on (A) IAAS values, (B) EE and (C) recovery efficacy of the PHB nanoparticles loaded with **3nInOAc** and **4nInOAc**. Significant results with 95% confidence.

Pareto chart (Figure 5A) and the ANOVA data (Table 7) disclosed that the stirring rate (Factor C) was the only parameter that significantly influenced the IAAS values and, consequently, the size of the polymeric nanoparticles. The IAAS value was decreased to 252.65 when the stirring rate was increased from 10,000 to 22,000 rpm. This influence can be noticed by comparing, for example, experiment 1 (IAAS = 510 ± 17) with experiment 5 (IAAS = 194 ± 1), and experiment 2 (IAAS = 532 ± 10) with experiment 6 (IAAS = 201 ± 17) (Table 6). This result corroborates previous studies since the increase of the stirring rate is commonly reported as the main factor responsible for the particle size reduction due to increase in the shear force during the emulsification process that favors the reduction of the droplet size of the organic phase and, consequently, the nanoparticle size.^36,56^

**Table 7:** ANOVA of the Effects of the Parameters Used in the Preparation of Nanoparticles on the IAAS Values of PHB Nanoparticle Suspensions Loaded with **3nInOAc** and **4nInOAc**

factor	SS^a^	Df^b^	MS^c^	*F*	prob > *F*
model	3.878 × 10^5^	7	55394.07	25.32	<0.0001
A	6518.67	1	6518.67	2.98	0.1080
B	4188.96	1	4188.96	1.91	0.1897
C	3.51 × 10^5^	1	3.51 × 10^5^	160.47	<0.0001
AB	10041.58	1	10041.58	4.59	0.0517
AC	7614.25	1	7614.25	3.48	0.0848
BC	3089.25	1	3089.25	1.41	0.2560
ABC	5234.21	1	5234.21	2.39	0.1459
curvature	9201.63	1	9201.63	4.21	0.0610
residual	28440.79	13	2187.75		
lack of fit	9.67	1	9.67	4.08 × 10^–3^	0.9501
pure error	28431.13	12	2369.26		
total SS	4.25 × 10^5^	21			

a SS = sum of squares.

b Df = degrees of freedom.

c MS = mean squares.

The change of the group [7-oxy-3-(3′,4′,5′-trimethoxyphenyl)coumarin] in the nonperipheral or peripheral position on the indium(III) Pc (Factor A) did not significantly influence the polymeric nanoparticle size (Figure 5A and Table 7), which can also be noticed if comparing experiment 3 (IAAS = 529 ± 4) with experiment 4 (IAAS = 523 ± 8) (Table 6). This result corroborates the theoretical calculations that showed an insignificant difference between the surface areas (1.5%) and volumes (1.4%) of **3nInOAc** and **4nInOAc** (Table 5).

The PS mass (Factor B) did not significantly influence the polymeric nanoparticle size (Figure 5A and Table 7). As each formulation behaves in a unique way concerning the nanoparticle preparation parameters, researchers have reported different results regarding the influence of the PS mass. In the preparation of PLGA polymeric nanoparticles containing *m*-THPP porphyrin, da Silveira et al. observed a reduction in particle size when the PS mass was increased,^57^ as well as Sashmal et al. and Javadzadeh et al. reported this same significant influence in their work.^58,59^ However, in the preparation of PLGA nanoparticles loaded with bupivacaine, Zhang et al. did not observe any effect of this same parameter on the nanoparticles size.^60^ Thus, we corroborate the importance of evaluating the effect of parameters used in the nanoparticle preparation since a parameter will not necessarily influence the properties of different formulations in the same way.

### 3.3 Influence of Factors A, B, and C on the Entrapment Efficiency of the **3nInOAc** or **4nInOAc** into the PHB Nanoparticles

The EE (%EE) of the **3nInOAc** into the PHB nanoparticles ranged from 51 ± 3% (Exp. 7) to 86 ± 2% (Exp. 1), while the efficiency for entrapping of the **4nInOAc** ranged from 46 ± 4% (Exp. 8) to 88 ± 3% (Exp. 2) (Table 6). The change of the [7-oxy-3-(3′,4′,5′-trimethoxyphenyl)coumarin] group (Factor A), from the nonperipheral (**3nInOAc**) to peripheral (**4nInOAc**) position, did not significantly influence the EE of the Pc into the PHB nanoparticles (Figure 5B and Table 8). Studies have shown that the physicochemical properties of the PS can interfere with the EE. For example, molecules with higher polarizability tend to diffuse more easily from the organic phase to the aqueous phase, decreasing the %EE, while PSs with greater surface area and volume tend to have lower %EE.^36,57^ Although the **4nInOAc** PS is more hydrophilic than the **3nInOAc** since its Log *P* and polarizability values are lower 15.3% and 1.27%, respectively, and its hydration energy is 2.8× higher than **3nInOAc** (Table 5), such differences were not significant to influence the EE of PSs.

**Table 8:** ANOVA of the Effects of the Parameters Used in the Preparation of Nanoparticles on the Entrapment Efficiency of the **3nInOAc** and **4nInOAc** into the PHB Nanoparticles

factor	SS^a^	Df^b^	MS^c^	*F*	prob > *F*
model	3253.91	7	464.84	27.74	<0.0001
A	75.46	1	76.46	4.56	0.0523
B	1610.76	1	1610.76	96.13	<0.0001
C	1346.82	1	1346.82	80.38	<0.0001
AB	1.58	1	1.58	0.094	0.7639
AC	63.70	1	63.70	3.80	0.0731
BC	126.13	1	126.13	7.53	0.0167
ABC	28.47	1	28.47	1.70	0.2151
curvature	28.29	1	28.29	1.69	0.2164
residual	217.84	13	16.76		
lack of fit	4.73	3	4.73	0.27	0.6153
pure error	213.11	12	17.76		
total SS	3500.03	21			

a SS = sum of squares.

b Df = degrees of freedom.

c MS = mean squares.

**Figure 6:**
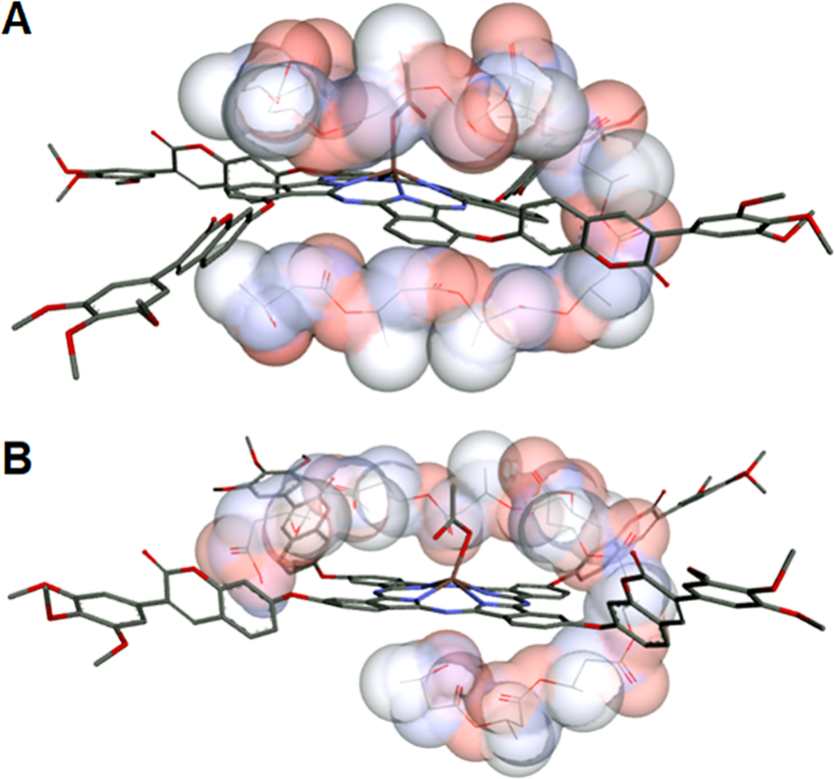
Superposition of the results of molecular docking by AutoDock Vina of the PHB molecule with the (A) **3nInOAc**—energy of −4.7 kcal/mol and (B) **4nInOAc**—energy of −4.3 kcal/mol.

Molecular docking disclosed that there is a small difference between the interaction energy of the **3nInOAc**-PHB polymer and **4nInOAc**-PHB polymer (Δ*G* binding—Table 5) since the energy of interaction was 8.5% lower for the **4nInOAc** PS when compared to the **3nInOAc** (Table 5). However, this result indicates a tendency for the **3nInOAc** Pc to have a greater affinity to PHB molecules (Δ*G* binding = −4.7 kcal/mol) compared with that of the **4nInOAc** Pc (Δ*G* binding = −4.3 kcal/mol) (Figure 6). The lower binding energy value is, the stronger the interaction between the polymer and the ligand.^21,61^ Thus, PHB polymeric nanoparticles loaded with **3nInOAc** tend to exhibit a higher EE in the polymer matrix. This outcome can be observed comparing, for example, experiment 5 (EE = 69 ± 3%) with experiment 6 (EE = 57 ± 1%) (Table 6). The lower interaction of **4nInOAc** with PHB molecules is associated with a change in the position of the tetrakis[7-oxy-3-(3′,4′,5′-trimethoxyphenyl)coumarin] substituent, from the nonperipheral position (**3nInOAc** PS) to the peripheral position (**4nInOAc** PS). This change (Factor A) caused a reduction in the EE of 3.73% even though it did not significantly influence the factorial design results. This decrease is also associated with the lower log *P* and polarizability values, as well as the higher hydration energy value of the **4nInOAc** PS if compared with the properties of the **3nInOAc** (Table 5). Molecular docking revealed that the PHB decamer interacts with the **3nInOAc** through the upper and lower part of the molecule without apparent differentiation, whereas the interaction of PHB with the **4nInOAc** molecule occurs preferentially through the upper part of Pc (Figure 6), evidencing the greater interaction of PHB with the **3nInOAc** (Table 5). Isomers 3 and 5 showed the highest values of interaction energy between **4nInOAc** isomers and PHB (−4.6 and −4.5 kcal/mol, respectively) (Table 5). Probably, the concentration of isomers 3 and 5 was lower than that of other structural isomers of **4nInOAc**, decreasing the ability of **4nInOAc** to interact with PHB.

The increase in the stirring rate (Factor C) caused a reduction (18.3%) in the EE (Figure 5B and Table 8). This effect can be observed, for example, comparing the experiment 1 (EE = 86 ± 2%) with the experiment 5 (EE = 69 ± 3%) and the experiment 2 (EE = 88 ± 3%) with the experiment 6 (EE = 57 ± 1%) (Table 6), in which there is variation only in Factor C. As previously discussed, the increase in the stirring rate leads to reduction of the particles size, an aspect that favors the diffusion of the PS from the organic phase to the aqueous solution, decreasing the EE of the Pc in the PHB nanoparticle.^45^

The increase of the PS mass (Factor B) was the main parameter that influenced the EE (Figure 5B and Table 8), decreasing the entrapment of 20.07%. This effect can be observed comparing experiment 1 (EE = 86 ± 2%) with experiment 3 (EE = 62 ± 4%) and experiment 2 (EE = 88 ± 3%) with experiment 4 (EE = 60 ± 4%) (Table 6), in which only Factor B is varied. This result suggests that the polymeric nanoparticles is capable to encapsulate a specific amount of PS since the increase of the Pc mass into the droplets of organic phase with high specific surface area favors the diffusion of the PS from the organic phase to the aqueous phase. The increase of Pc mass increases the organic phase viscosity and, consequently, decreases the coacervation rate of the organic phase droplets, favoring the diffusion of the PS from the organic phase to the aqueous phase and the decrease of the PS entrapment into the nanoparticle.^62,63^ Yamakawa et al. reported the same effect when increasing the concentration of the encapsulated compound, showing that the %EE increased up to a certain concentration and then tended to decrease.^64^ Govender et al. also observed a reduction in the EE when increasing the mass of procaine in PLGA nanoparticles.^65^

The simultaneous increase of the stirring rate and PS mass (binary factor BC) increased the EE by 5.61%, disclosing that the BC binary effect is significant in the EE of the evaluated Pc (Figure 5B and Table 8). Despite the individual effect of each factor B and C causing a reduction in %EE, the BC binary factor increases the entrapment of **3nInOAc** and **4nInOAc** into the PHB polymeric nanoparticles. The increase of the stirring rate simultaneously with the increase of the organic phase viscosity may be favoring the coacervation of the PHB on the nanodroplet surface of the organic phase, increasing the EE of studied compounds.

### 3.4 Influence of Factors A, B, and C on the Recovery Efficacy of the PHB Nanoparticles Loaded with **3nInOAc** or **4nInOAc**

The recovery efficacy of **3nInOAc**-loaded nanoparticles ranged from 48 ± 19% (Exp. 7) to 87 ± 4% (Exp. 1), while for **4nInOAc**-loaded nanoparticles ranged from 39 ± 1% (Exp. 8) to 79 ± 13% (Exp. 2) (Table 6).

The nanoparticle size is directly related to the recovery efficacy since the sedimentation rate of the particles in a centrifugal field is proportional to the square of the particles diameter. Therefore, particles with smaller sizes are expected to have a lower recovery efficacy than particles with larger sizes.^36^

The effect of Factor C in causing a reduction of 14.54% in the recovery efficacy (Figure 5C) corroborates the influence of this same factor to decrease 18.3% of the particle size (Figure 5A) since the increase in the stirring rate reduces the particle size and consequently also decreases their recovery. The ANOVA data (Table 9) corroborated that the Factor C significantly influenced the recovery efficacy, as shown in the Pareto chart (Figure 5C).

**Table 9:** ANOVA of the Effects of the Parameters Used in the Preparation of Nanoparticles on the Recovery Efficacy of the PHB Nanoparticles Loaded with **3nInOAc** and **4nInOAc**

factor	SS^a^	Df^b^	MS^c^	*F*	prob > *F*
model	4584.41	7	654.92	9.27	0.0003
A	796.08	1	796.08	11.27	0.0051
B	2413.77	1	2413.77	34.17	<0.0001
C	845.57	1	845.57	11.97	0.0042
AB	153.96	1	153.96	2.18	0.1637
AC	184.78	1	184.78	2.62	0.1298
BC	0.55	1	0.55	7.72 × 10^–3^	0.9313
ABC	189.70	1	189.70	2.69	0.1252
curvature	702.01	1	702.01	9.94	0.0076
residual	918.23	13	70.63		
lack of fit	190.21	3	190.21	3.14	0.1020
pure error	728.02	12	60.67		
total SS	6204.65	21			

a SS = sum of squares.

b Df = degrees of freedom.

c MS = mean squares.

The change of position of the substituent group [7-oxy-3-(3′,4′,5′-trimethoxyphenyl)coumarin] from nonperipheral (**3nInOAc** PS) to peripheral (**4nInOAc** PS) on the indium(III) Pc (Factor A) caused a decrease of 12.03% in the recovery efficacy (Figure 5C and Table 9), disclosing that PHB nanoparticles loaded with **3nInOAc** showed better recovery efficacy than **4nInOAc**-loaded particles. Likewise, the recovery of nanoparticles decreased by 24.56% (Figure 5C and Table 9) when the PS mass was increased from 0.15 to 0.28 mg (Factor B). Govender et al. observed the same effect and suggested that it can be attributed to the destabilization of the nanoparticles suspension due to the higher concentration of compounds on the nanoparticles surface.^62^ Feng et al. also reported the relation between the increase in the PS mass and recovery efficacy.^63^ However, as each formulation has its particularity, other studies also showed that this parameter did not influence the recovery efficacy of nanoparticles.^65^

Although Factor A did not significantly influence the IAAS values, the 2^3^ factorial design disclosed that the change from the nonperipheral to peripheral position of the tetrakis[7-oxy-3-(3′,4′,5′-trimethoxyphenyl)coumarin] substituent caused a reduction of 34.43 in the IAAS value, suggesting a decrease in the nanoparticles size. This result suggests that the smaller volume of the **4nInOAc** and the smaller interaction of the **4nInOAc** molecule with the PHB favor the generation of smaller diameter nanoparticles and, consequently, a reduction in the recovery efficacy. In this context, the higher concentration of isomer 1 in the mixture of the structural isomers could reduce the recovery efficacy of **4nInOAc** since this isomer has the smallest molecular volume and a small interaction energy with PHB.

### 3.5 Albumin and Trp Photooxidation

#### 3.5.1 Morphology and Evaluation of the Nanoparticulate Formulations Used in the Photooxidation Experiment

Considering the results of the individual and binary effects of parameters used in nanoparticle preparation on the nanoparticulate properties, a smaller PS mass with a lower stirring rate should be used to optimize the EE. However, this procedure would increase the size of nanoparticles and would decrease the percentage loading (mass of encapsulated PS by lyophilized nanoparticle mass), which would hinder the photodynamic efficiency since the permanence of the nanoparticle in the bloodstream and the concentration of the generated oxygen singlet would be decreased. Therefore, the formulations of experiments 5 for the **3nInAOc**-loaded PHB nanoparticle and 6 for the **4nInOAc**-loaded PHB nanoparticle were chosen and used for the photooxidation experiments since these experiments presented the best correlation between the nanoparticle size, EE and the recovery efficacy of the particles.

**Figure 7:**
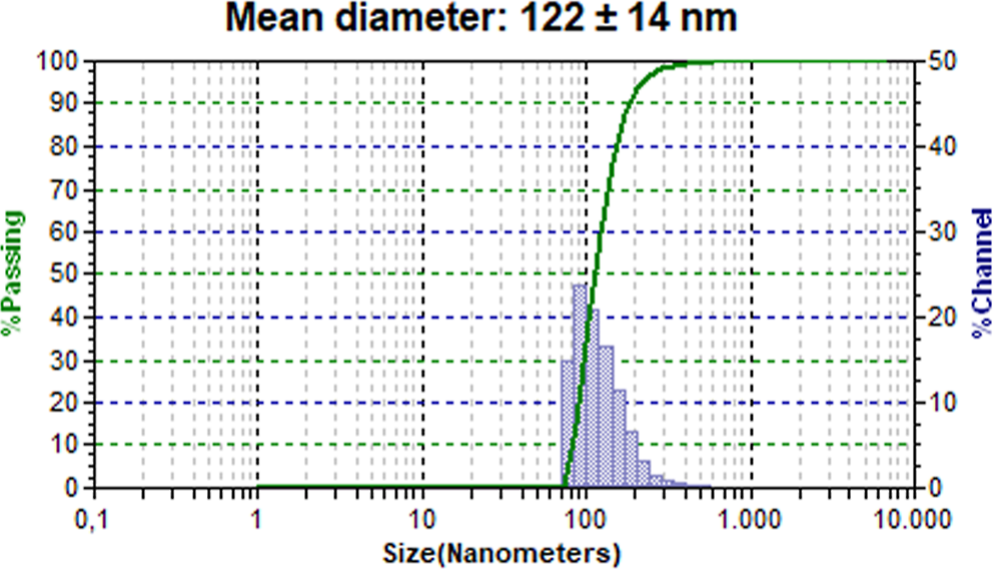
Size distribution of PHB nanoparticles loaded with **4nInOAc** prepared according to experiment 6 of the 2^3^ factorial design, respectively.

The experiment 5 was chosen for the photooxidation experiment that it was performed with the **3nInOAc** Pc due to the lowest IAAS value (194 ± 1) and satisfactory outcomes of entrapment and recovery efficacy (EE = 69 ± 3% and ER = 86 ± 4%), mainly characterized by particles with a mean diameter smaller than 200 nm (Figure 4A) and a mean zeta potential of −15.9 ± 2.9 mV. The negative zeta-potential value is typical of the carboxylic groups present on the nanoparticle surface and can also be influenced by the PVA molecules present on the surface of the PHB nanoparticles.^66^ For the nanoparticles loaded with **4nInOAc**, the formulation that presented the best correlation between the evaluated properties was experiment 6 (AIEA = 201 ± 17, EE = 57 ± 1%, and ER = 50 ± 5%), being characterized with a mean zeta potential of −18.3 ± 2.2 mV and by particles with a mean diameter of 122 ± 14 nm. This formulation showed that 93.7% of the particles had diameters between 86 and 204 nm (Figure 7).

**Figure 8:**
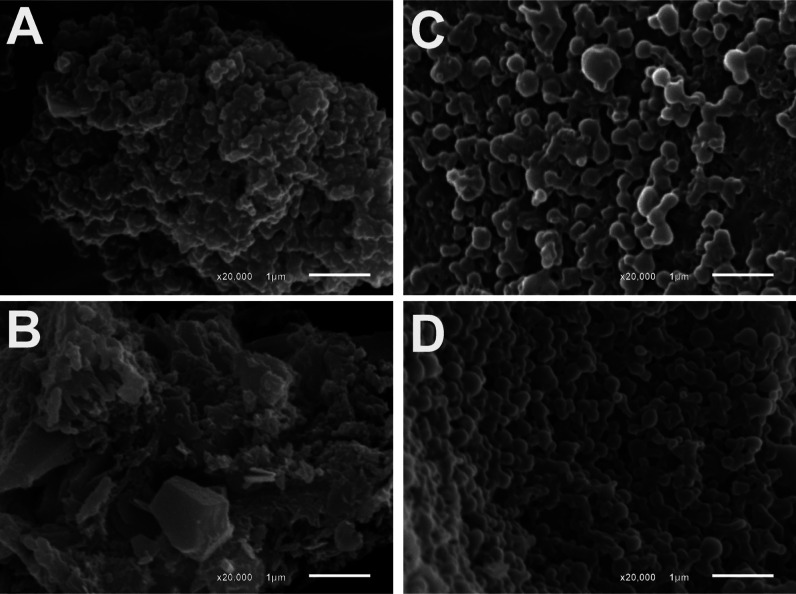
Scanning electronic microscopy images of (A) free Pc **3nInOAc**, (C) **3nInOAc** loaded PHB nanoparticle, (B) free Pc **4nInOAc**, and (D) **4nInOAc**-loaded PHB nanoparticle.

SEM images (Figure 8) showed a relatively spherical morphology and homogeneous size distribution of PHB nanoparticles containing the PSs (Figure 8C,D) compared to the free PS in the absence of the polymer (Figure 8A,B), which showed irregular morphologies and smaller size than the polymeric nanoparticles, indicating that the PSs were encapsulated in the polymeric matrix of PHB.

#### 3.5.2 Albumin and Trp Photooxidation Using the PHB Nanoparticles Loaded with **3nInOAc** and **4nInOAc**

**Figure 9:**
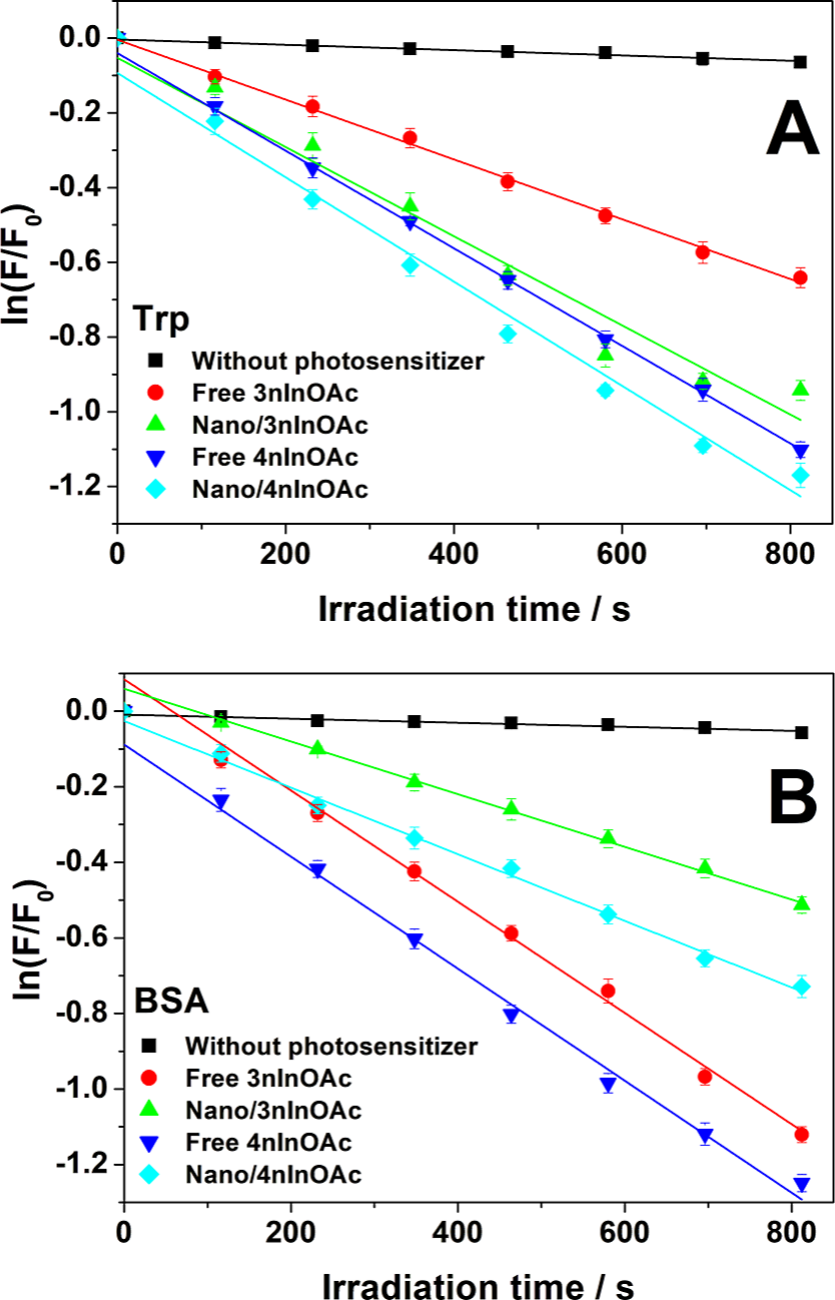
Kinetic graphic of the ln (*F*/*F*_0_) versus irradiation time for the photooxidation of (A) Trp and (B) albumin, (■) in the absence of PS—control experiment, (●) with free **3nInOAc**, (▲) with encapsulated **3nInOAc**, (▼) with free **4nInOAc**, and (◆) with encapsulated **4nInOAc**.

Photooxidation results indicate that there was a small photodegradation of Trp in the absence of a PS due to the laser energy (control experiment) (Figure 9A). However, the Trp photooxidation rate increased by 10 and 17× in the presence of free **3nInOAc** and **4nInOAc**, respectively, if compared with the photooxidation caused by only the laser energy (Table 10). The significant difference between the free Pc to cause the Trp photooxidation can be attributed to the lower hydrophobicity of **4nInOAc** (Log *P* = 12.20—Table 5) compared to the **3nInOAc** (Log *P* = 14.41—Table 5) since the reduction of the hydrophobicity decreases the Pc aggregate state and, consequently, favors the generation of singlet oxygen and the greater photodynamic efficiency.^36^ The intensity absorbance of the free **4nInOAc** spectrum profile was decreased 3.3× when the PS was solubilized in PBS solution, while the intensity of the free **3nInOAc** spectrum was reduced for 2.8× (Figure 2), as well as the fluorescence intensity of the free **4nInOAc** decreased 7.0× while the intensity of the **3nInOAc** reduced 3.9×. These results suggest that **4nInOAc** was in a higher state of aggregation than that was the **3nInOAc**. Researchers have showed that the hydrophobic PSs tend to form aggregates in aqueous medium, decreasing their bioavailability, the ability to absorb light, and consequently, to generate ROS.^24,36,49^ Researchers also reported that the singlet oxygen quantum yield was higher for the **3nInOAc** than that for the **4nInOAc** in DMSO.^25^ These results could suggest that **3nInOAc** would be more efficient in photooxidizing Trp, but **4nInOAc** was more efficient.

**Table 10:** Trp Photooxidation Rate Constant by the Free or Encapsulated **3nInOAc** and **4nInOAc** Solutions (15 μmol/L), Using 104 mW Laser Power^a^

compound	photooxidation rate constant/s^–1^	
without PS (control)	(0.8 ± 0.1) × 10^–4c^	
	free	encapsulated
**3nInOAc**	(8.0 ± 1.0) × 10^–4b^	(12.7 ± 1.1) × 10^–4a^
**4nInOAc**	(13.7 ± 2.5) × 10^–4a^	(14.7 ± 0.5) × 10^–4a^

a The values followed by the same letter do not differ from each other by the Tukey Test at a 5% significance level.

Researchers also showed that the photodegradation quantum yield was higher for the **4nInOAc** than that for the **3nInOAc** in DMSO.^25^ Considering that both photochemical processes (the singlet oxygen generation and PS photodegradation) occur simultaneously, the major photodegradation of the **4nInOAc** favored the lesser efficiency of this compound to generate the singlet oxygen in DMSO.^25^ However, in this work, the Trp photooxidation was monitored in PBS solution, where the aqueous medium caused the major aggregation of the free **4nInOAc** than in the free **3nInOAc** (Figure 2). It is necessary to consider that **4nInOAc** is a mixture of eight structural isomers (Figure 3). Each isomer can show a different behavior in aqueous medium due to its hydrophobicity, but in general the mixture of isomers of **4nInOAc** was lesser hydrophobic (Table 3) than that was the **3nInOAc** (Table 5). Researchers showed that the photodegradation rate is decreased when the free PS is aggregated.^24^ Therefore, the lower hydrophobicity of **4nInOAc** (Tables 3 and 5) and the reduction in the photodegradation process of free **4nInOAc** favored the best photodynamic results for this compound in the Trp photooxidation. The PHB nanoparticles loaded with **3nInOAc** or **4nInOAc** were 16× and 18×, respectively, faster to photooxidate the Trp than that was the control experiment (Table 10). The rate constant also increased 1.6× when the encapsulated **3nInOAc** was used in the Trp photooxidation if compared with the free **3nInOAc** (Table 10). These results are very clear comparing the decrease profile of the Trp fluorescence intensity since the inclination angle of the light blue straight line (Nano/**4nInOAc**) is major than that the green straight line (Nano/**3nInOAc**), or the green straight line (Nano/**3nInOAc**) with the red straight line (Free **3nInOAc**) (Figure 9A). The encapsulation of the **3nInOAc** into the PHB nanoparticle reduced its molecular aggregation state, favoring the singlet oxygen generation, and consequently, a better photodynamic efficiency in the Trp photooxidation if compared to free **3nInOAc**. These results corroborate with studies that have shown a trend of encapsulation improves the PS performance in the biomolecule photooxidation.^21,67,68^ However, there is no significant difference between the rate constant of the Trp photooxidation performed with the encapsulated **4nInOAc** if compared to the free **4nInOAc**. Molecular docking (Figure 6) showed that **4nInOAc** has a lower interaction than **3nInOAc** with the PHB molecules (Table 5). This result suggests that the encapsulation of the **4nInOAc** did not significantly reduce the aggregation state of the **4nInOAc** as occurred with the encapsulated **3nInOAc**, resulting in insignificant differences between the encapsulated and free **4nInOAc** to photooxidate the Trp. Researchers also showed that the encapsulation reduced significantly the PS photodegradation,^24^ decreasing the influence of this photochemical process on the Trp photooxidation by encapsulated PS. Therefore, these results showed that the interaction between the PS and the polymer is important to reduce the aggregation state of the encapsulated PS and improve its efficiency to photoxidate Trp.

The laser energy also caused a small BSA photooxidation without the presence of the PS (Figure 9B), but in the presence of free **3nInOAc** and **4nInOAc**, the reaction rate increased 23× and 25×, respectively (Table 11). However, there was no significant difference between the rate constant of BSA photooxidation using the free **3nInOAc** and **4nInOAc** (Table 11), as it was observed in the Trp photooxidation (Table 10). This result was corroborated with the decrease profile of the BSA fluorescence intensity since the angle of the red straight line (Free **3nInOAc**) is the same of the dark blue straight line (Free **4nInOAc**) (Figure 9B).

**Table 11:** BSA Photooxidation Rate Constant by the Free or Encapsulated Photosensitizer Solutions (15 μmol/L), Using 104 mW Laser Power^a^

compound	photooxidation rate constant/s^–1^	
without PS (control)	(0.6 ± 0.2) × 10^–4c^	
	free	encapsulated
**3nInOAc**	(14.0 ± 2.6) × 10^–4a^	(6.5 ± 0.7) × 10^–4b^
**4nInOAc**	(15.3 ± 0.6) × 10^–4a^	(9.0 ± 0.1) × 10^–4b^

a The values followed by the same letter do not differ from each other by the Tukey Test, at a 5% significance level.

**Figure 10:**
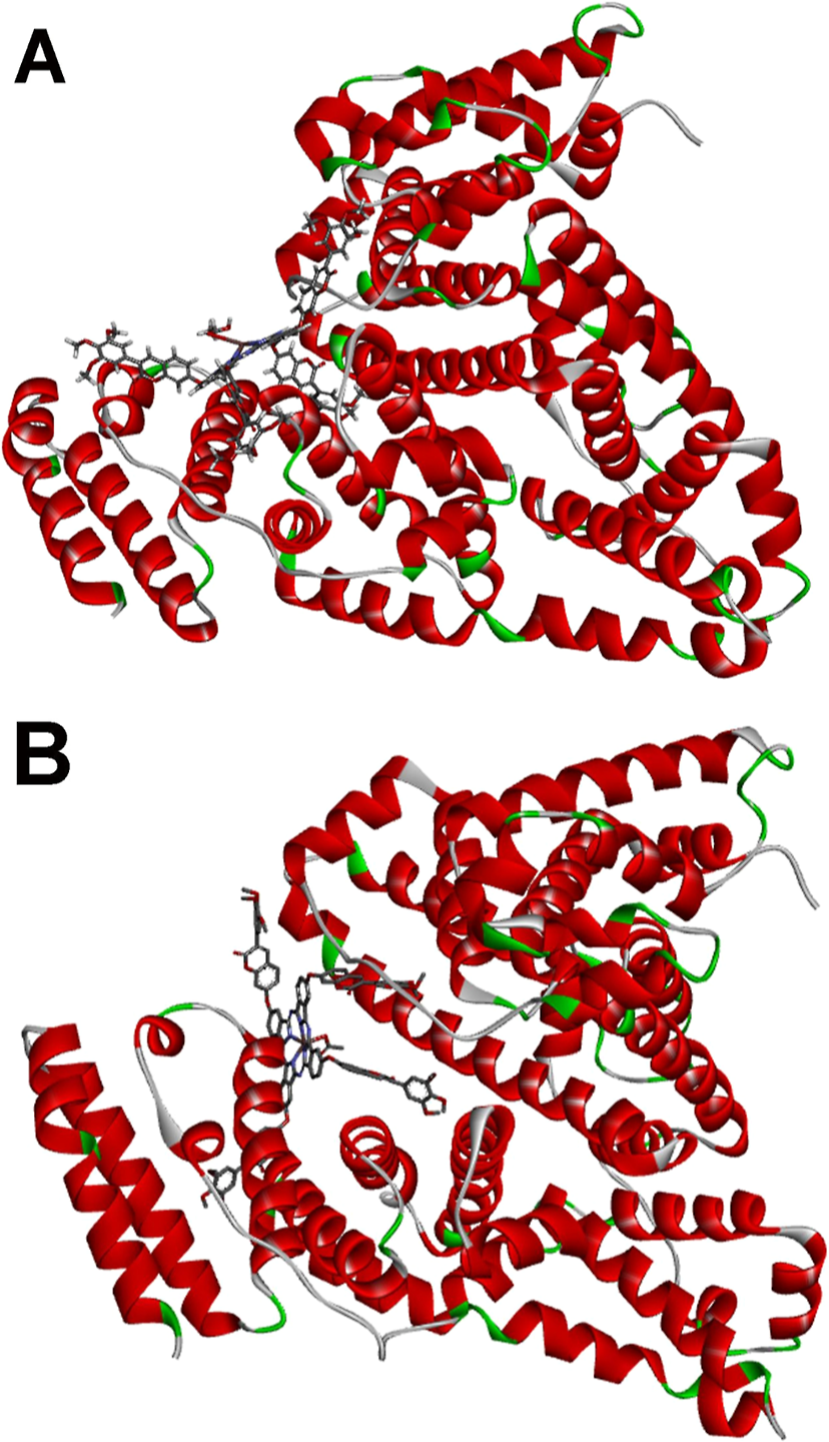
Superposition of the results of molecular docking of the (A) **3nInOAc** and (B) **4nInOAc** with BSA by AutoDock Vina.

The molecular docking disclosed that the energy (Δ*G*_binding_) of the BSA-**3nInOAc** interaction presented a value of −11.1 kcal/mol (Figure 10A) while the BSA-**4nInOAc** interaction was −12.3 kcal/mol (Figure 10B). Our research group has already shown that the greater the interaction between the protein and Pc, it may be difficulties for the PS molecule to transfer energy to the oxygen molecules, hindering its photodynamic efficiency, since the PS would be less available to react with ground state oxygen present in the medium.^21^ In this context, the higher concentration of isomers 2, 3, and 4 in the mixture of structural isomers could favor the reduction of singlet oxygen generation since these isomers showed the highest interaction energy with BSA (Table 4). Molecular docking results showed a tendency toward a greater interaction of BSA with the **4nInOAc** than with the **3nInOAc**. However, the difference between the energies was not expressive, corroborating the nonsignificant difference between the photodynamic efficiencies of BSA photooxidation by free Pc (Table 11). It should also be noted that molecular docking revealed an interaction of **3nInOAc** with the outermost region of BSA (Figure 10A), while the **4nInOAc** molecule showed a more internal interaction (Figure 10B). Probably, the highest aggregation state of **4nInOAc** favored this compound to interact in more internal regions of the protein, while **3nInOAc** was able to interact with the outermost region of the protein. This fact could favor some of the Pc to photooxidize the protein since researchers have shown that BSA has two Trp molecules localized in two different subdomains, being one in the protein hydrophobic fold and second in a more superficial region of the protein. However, it was not observed the difference between the **3nInOAc** and the **4nInOAc** to photooxidate the BSA. Therefore, the position change of the substituent group [7-oxy-3-(3′,4′,5′-trimethoxyphenyl)coumarin] on the indium(III) Pc did not influence the BSA photooxidation by free Pc.

The encapsulated **3nInOAc** and **4nInOAc** were able to photooxidize 10.8× and 15× faster the BSA than the control experiment (Table 11). However, a decrease of 54% and 41% was observed in the photooxidation rate of BSA when the **3nInOAc** and **4nInAOc**, respectively, were encapsulated if compared with the photooxidation performed in the presence of free Pc (Table 11). It is likely that the interaction of the encapsulated PS molecules with the polymeric matrix hampers the PS interactions with the protein binding sites, decreasing the photodynamic efficiency of BSA photooxidation.^21^

Molecular docking showed that the interaction energy of the **3nInOAc**-PHB polymer was a little greater than that observed for the **4nInOAc**-PHB polymer interaction (Table 5). Probably, this small difference was not able to hamper the transfer energy of the excited **3nInOAc** to the oxygen molecular since the encapsulated **3nInOAc** presented the same photodynamic efficiency than the encapsulated **4nInOAc** in photooxidizing BSA. It is necessary to consider that the **4nInOAc** is a mixture of structural isomers. If the concentration of isomers 2, 3, and 4 was higher than those other isomers, probably the higher interaction energy of these isomers with PHB (Table 4) could hamper the energy transfer from excited **4nInOAc** to molecular oxygen, reducing the generation of singlet oxygen.

**Figure 11:**
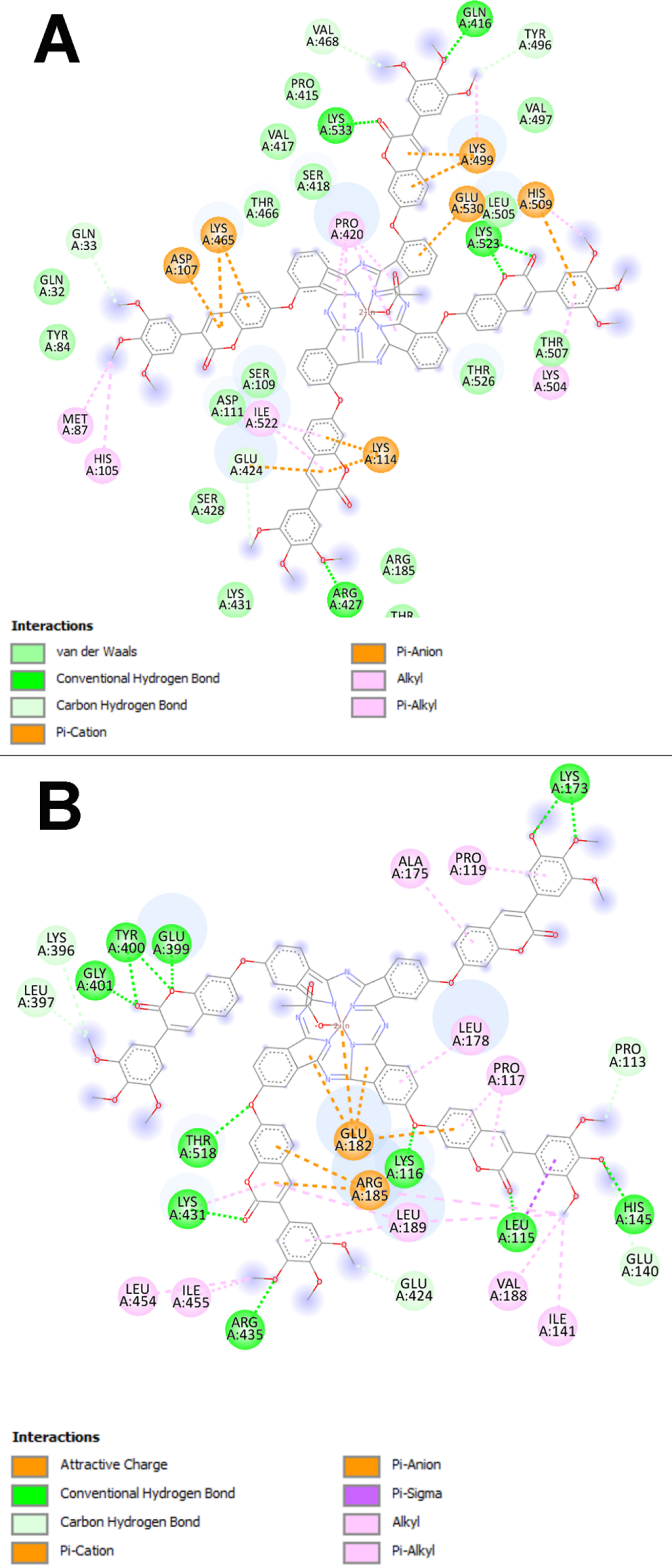
2D interaction map between (A) **3nInOAc** and (B) **4nInOAc** with BSA.

Analyzing the 2D interaction map between the **3nInOAc** and **4nInOAc** with BSA, both complexes (**3nInOAc**-BSA and **4nInOAc**-BSA) have essentially the same types of interactions, except by a Pi-Sigma and attractive charge marked by the **4nInOAc** complex with Leu115 and Glu182, respectively (Figure 11). Mainly, the attractive charge interaction between Glu182 and the Pc core can explain the higher docking energy between **4nInOAc** and BSA (Figure 11B). The **4nInOAc** showed a greater number of conventional hydrogen bond (Figure 11B) than the **3nInOAc** with BSA (Figure 11A), corroborating the characteristic of being a less hydrophobic molecule. The result also showed that the **3nInOAc** macrocycle containing four isoindole units maintained a similar structure (Figure 11A) to the **4nInOAc** macrocycle (Figure 11B), even though both molecules have different interactions with BSA. The acetate group linked to the central metallic atom is spatially oriented toward one of the isoindole units, tensioning the opposite side of the Pc nucleus, resulting in this apparent asymmetry between the four isoindole units.

### 3.6 In Vitro Photodynamic Inactivation of *S. aureus*

The microorganism susceptibility to photodynamic reaction may vary according to their structural characteristics and resistance mechanisms.^14,69,70^ Thus, microbiological assays were performed using two distinct strains of *S. aureus* bacteria, one methicillin-susceptible (MSSA—ATCC 29213) and another methicillin-resistant (MRSA—ATCC 43300). The success of microorganism inactivation by APDT depends on the treatment parameters, considering the type and concentration of the PS, the incubation time, and dose of irradiation. The parameters applied in this work were established according to previous studies.^71−73^

**Figure 12:**
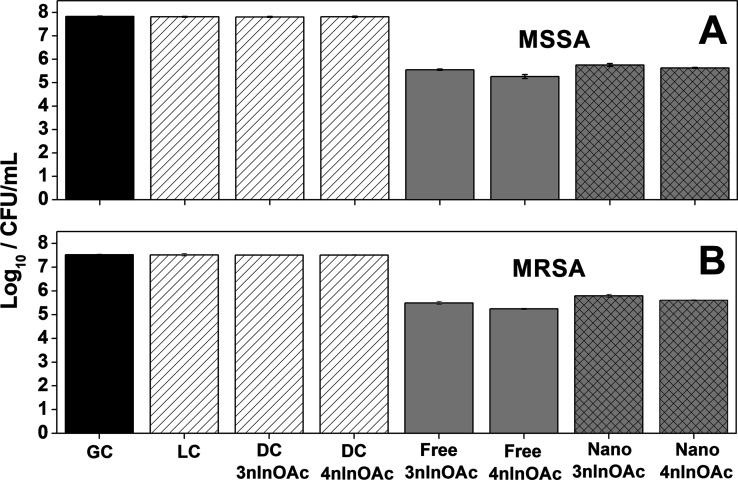
Cell viability of (A) MSSA—ATCC 29213 and (B) MRSA—ATCC 43300 after photodynamic inactivation assays treated with free and encapsulated 3nInOAc and 4nInOAc, including growth control (GC), light control (LC), and dark control (DC for **3nInOAc** and **4nInOAc**).

The results showed (Figure 12) that the laser light on the absence of PS (LC = light control) and the cytotoxicity effect of PSs without laser irradiation (DC/**3nInOAc** and DC/**4nInOAc** = dark control) did not cause any reduction on the viability of the MSSA or MRSA strains. However, a significant photocytotoxicity effect was observed when the MSSA bacteria culture was treated with the free and encapsulated **3nInOAc** and **4nInOAc**, since the Log_10_/CFU/mL decreased from 2.08 to 2.57 logs in the viability of the bacteria if compared to the growth control experiment (GC—absence of PS or light irradiation) (Table 12).

**Table 12:** Cell Viability of the *S. aureus* MSSA—ATCC 29213 and MRSA—ATCC 43300 after APDT Treatments^a^

S. aureus	treatments	log_10_/CFU/mL	reduction/log_10_	reduction/%
MSSA (ATCC 29213)	GC	7.83 ± 0.01^a^		
	LC	7.82 ± 0.02^a^	0.02 ± 0.003	3.68 ± 0.77
	DC/**3nInOAc**	7.81 ± 0.02^a^	0.02 ± 0.01	5.40 ± 1.86
	DC/**4nInOAc**	7.82 ± 0.02^a^	0.02 ± 0.01	3.71 ± 1.99
	free **3nInOAc**	5.55 ± 0.03^c^	2.28 ± 0.02	99.47 ± 0.02
	free **4nInOAc**	5.26 ± 0.09^d^	2.57 ± 0.07	99.73 ± 0.04
	nano/**3nInOAc**	5.75 ± 0.06^b^	2.08 ± 0.07	99.16 ± 0.15
	nano/**4nInOAc**	5.63 ± 0.02^c^	2.20 ± 0.03	99.37 ± 0.04
MRSA (ATCC 43300)	GC	7.52 ± 0.02^e^		
	LC	7.52 ± 0.05^e^	0.00 ± 0.03	0.51 ± 6.02
	DC/**3nInOAc**	7.51 ± 0.01^e^	0.01 ± 0.01	2.24 ± 2.94
	DC/**4nInOAc**	7.51 ± 0.02^e^	0.01 ± 0.01	2.40 ± 2.28
	free **3nInOAc**	5.50 ± 0.06^g^	2.03 ± 0.07	99.05 ± 0.17
	free **4nInOAc**	5.24 ± 0.02^h^	2.28 ± 0.04	99.47 ± 0.05
	nano/**3nInOAc**	5.79 ± 0.06^f^	1.73 ± 0.08	98.13 ± 0.36
	nano/**4nInOAc**	5.61 ± 0.02^g^	1.92 ± 0.04	98.78 ± 0.11

a The values followed by the same letter do not differ from each other by the Tukey Test, at a 5% significance level.

The free **4nInOAc** was 1.13× more efficient in reducing the viability of MSSA bacteria compared to free **3nInOAc**, as well as the encapsulated **4nInOAc** was 1.06× more efficient than the encapsulated **3nInOAc** (Table 12). The greatest decrease in the viability of MSSA bacteria was obtained with the free **4nInOAc** in the presence of light dose since the viability was reduced of 2.57 logs, equivalent to 99.73% of the initial colonies. The lower hydrophobicity of **4nInOAc** reduces the tendency of molecular aggregation and, consequently, favors a greater photodynamic efficiency of free **4nInOAc** if compared with free **3nInOAc**, as was also observed in the photooxidation of Trp (Table 10). The reduction of the photodegradation rate caused by the PS aggregation also favored the action of the free **4nInOAc**, considering that the photodegradation quantum yield was major for the **4nInOAc** than that was for the **3nInOAc**.^23^

It is noteworthy that free Pc showed greater photodynamic efficiency than the PHB nanoparticles loaded with the Pc since the free **3nInOAc** was 1.10× more efficient than the encapsulated **3nInOAc**, while the free **4nInOAc** was 1.17× more efficient than encapsulated **4nInOAc** (Table 12). However, it should be noted that the encapsulation of the PSs caused a significant reduction in cell viability of MSSA bacteria closed to those obtained with free PSs (Figure 12A), indicating that the encapsulation of the **3nInOAc** and **4nInOAc** into the PHB polymeric nanoparticles does not compromise the photodynamic action of the PS and still can be a good alternative for in vivo treatments because the encapsulation favors the intravenous administration of the hydrophobic compounds.^74^

There was also a significant reduction in the MRSA bacteria viability when it was treated with each of the free and encapsulated PS (**3nInOAc** or **4nInOAc**) since the viability decreased from 1.73 to 2.28 logs (Table 12), showing that APDT can be effective in inactivating AMR strains.

The free **4nInOAc** was 1.12× more efficient in reducing the viability of MRSA bacteria compared to free **3nInOAc**, as well as encapsulated **4nInOAc** was 1.11× more efficient than the encapsulated **3nInOAc** in reducing bacteria viability. The greatest reduction in the viability of MRSA bacteria was obtained when the culture was irradiated in the presence of free **4nInOAc**, as the viability was reduced to 2.28 logs, equivalent to 99.47% of the initial colonies, due to the lower hydrophobicity of **4nInOAc** and the reduction in the photodegradation process of free **4nInOAc**, as was also observed in the MSSA strain assays.

Both free PSs showed greater photodynamic action in inhibiting the viability of MRSA bacteria than the encapsulated compound, since the free **3nInOAc** was 1.17× more efficient than encapsulated **3nInOAc** in reducing cell viability, while free **4nInOAc** was 1.19× more efficient than encapsulated **4nInOAc**. Thus, the encapsulation of PSs (**3nInOAc** and **4nInOAc**) reduced the antimicrobial efficiency against both strains studied when compared to that of free PSs. This result may have been caused by the negative surface charge of the PHB nanoparticles (−15.9 ± 2.9 and −18.3 ± 2.2 mV, for nanoparticles loaded with **3nInOAc** and **4nInOAc**, respectively). Negative charges can make it difficult for nanoparticles to interact with the bacterial cell membrane, which also has negative charges on its surface.^75^ Studies have reported that nanoparticles with cationic surface favor the contact of nanoparticle surface with the bacterial membrane causing osmotic damage by increased permeability of bacterial membrane.^76−78^

Another parameter that must be considered is the PS release time by the polymer matrix in the incubation step (60 min), which may not have been adequate in the investigated treatment. However, the encapsulated **4nInOAc** obtained a greater antimicrobial action than the encapsulated **3nInOAc**, corroborating the results obtained in the photooxidation of Trp and BSA (Figure 9) due to the lower hydrophobicity of the **4nInOAc** molecule (Table 5) that favors its concentration in the surface area of the PHB nanoparticles (Figure 6). In this context, the higher concentration of the isomers 1, 3, 4, and 8 in the mixture of the structural isomers could favor the better results showed by **4nInOAc** in the inactivation of MSSA and MRSA bacteria if compared that the results obtained for the **3nInOAc**.

Although the encapsulation of **3nInOAc** and **4nInOAc** in the polymeric matrix of PHB nanoparticles caused a reduction in the photodynamic efficiency of the inactivation of MSSA and MRSA bacteria in vitro, for in vivo assays and clinical trials this reduction may be irrelevant in view of the inherent benefits of encapsulation. Therefore, encapsulation should be considered because it protects the PS against enzymatic degradation, increases stability and optimization in the administration of PSs, also increasing their bioavailability, in addition to allowing successive irradiation actions depending on the excretion rate of nanoparticles by the organism.^36,79^

A statistically significant difference was observed for the viability of the MSSA and MRSA strains (Table 12) treated with the free or encapsulated Pc (**3nInOAc** or **4nInOAc**) since the viability of the MSSA bacteria was greater than that of the MRSA bacteria. This result suggests that in vitro methicillin-sensitive *S. aureus* is moderately more susceptible to APDT by the studied Pc and on the established conditions in the experiment.

Some studies also have reported a reduction in the inhibition of the viability of *S. aureus* bacteria similar to what was found in this work (∼2 logs).^74,76,80^ However, despite the results indicating the photodynamic effect of Pc **3nInOAc** and **4nInOAc**, this antimicrobial activity of the studied compounds is considered moderate as the log reductions obtained were below the recommended level for a methodology to be considered antimicrobial, which is a reduction of more than 3 logs in the viability of planktonic cells of the microorganism.^81,82^ However, the antimicrobial effect of these PSs can be improved by coating PHB nanoparticles with chitosan, making the surface of the particles positive.^83^

## 4 Conclusions

The polymeric nanoparticles loaded with Pc, obtained according to the 2^3^ factorial design, showed a spherical morphology with a mean diameter smaller than 200 nm. PHB nanoparticles loaded with **3nInOAc** Pc showed an EE of 69 ± 3%, a recovery efficacy of 86 ± 4%, and a zeta potential of −15.9 ± 2.9 mV, while the formulations with **4nInOAc** were characterized with an EE of 57 ± 1%, a recovery of 50 ± 5%, and a zeta potential of −18.3 ± 2.2 mV. Probably, the structural isomers with lower interaction energy between **4nInOAc** and PHB favored the lower EE of the **4nInOAc** in the PHB nanoparticle compared to the EE of the **3nInOAc**.

Since each formulation behaves in a unique way regarding the nanoparticle preparation parameters, the factorial design allowed us to estimate the effects of the position (peripheral or nonperipheral) of the tetrakis[7-oxy-3-(3′,4′,5′-trimethoxyphenyl)coumarin] substituent in the indium(III) Pc on the nanoparticulate properties. The position change of the coumarin group from nonperipheral (**3nInOAc**) to peripheral (**4nInOAc**) in the Pc, as well the stirring rate and the PS mass, caused a decrease on the recovery efficacy of the PHB nanoparticle. Although the position of the coumarin group was not a significant factor to cause some effect on the size and EE of the polymeric nanoparticles, the theoretical calculations indicated the difference between the physicochemical properties of studied Pc. Molecular docking disclosed the **3nInOAc** has a greater affinity to PHB molecules than the **4nInOAc**, showing that the position of the coumarin group in the indium(III) Pc can influence the localization of the PS into the polymeric matrix. The stirring rate was the only parameter responsible for decreasing the PHB nanoparticle size, although other studies have shown that the drug mass can change the nanoparticle size. The PS mass and the stirring rate reduced the EE of **3nInOAc** and **4nInOAc**. However, a synergic effect increased the PS entrapment when the stirring rate and PS mass were increased simultaneously. Factorial design outcomes corroborated the importance of evaluating the effect of parameters used in the nanoparticle preparation since a parameter will not necessarily influence the properties of different formulations in the same way.

The lower hydrophobicity of the **4nInOAc** and the reduction of the **4nInOAc** photodegradation showed to be relevant in the Trp photooxidation assays since the **4nInOAc** was more efficient to photooxidate the Trp and *S. aureus* bacteria than it was the **3nInOAc**.

The Trp photooxidation also disclosed that the encapsulation of **3nInOAc** improved its photodynamic efficiencies if compared to the free **3nInOAc** due to the reduction of the aggregation state of the PS. The same result was not observed for free and encapsulated **4nInOAc**. Molecular docking showed that **4nInOAc** has a lower interaction with the PHB than the **3nInOAc**. Probably, this smaller interaction should hinder a good disaggregation of the **4nInOAc** molecules into the PHB nanoparticles, influencing the photodynamic result of the encapsulated compound compared with that of the free **4nInOAc** and with that of the encapsulated **3nInOAc**. However, the encapsulation decreased the PS interaction with the protein and, consequently, the photodynamic efficiency, decreasing the photooxidation rate of BSA caused by the encapsulated **3nInOAc** and **4nInOAc** if compared with the results obtained by free compounds. The small difference of the interaction energy between the free **3nInOAc**-BSA and **4nInOAc**-BSA corroborated with the nonsignificant difference on the BSA photooxidation between the free PSs. Probably, the structural isomers with higher interaction energy between the **4NInAOc** and BSA, hampered the energy transfer from excited **4nInOAc** to molecular oxygen, reducing the generation of the singlet oxygen. Therefore, the results suggest that the nonperipheral or peripheral position of the [7-oxy-3-(3′,4′,5′-trimethoxyphenyl)coumarin] substituent groups on the indium(III) Pc was not influenced by the BSA photooxidation by free Pc.

The free and encapsulated **3nInOAc** and **4nInOAc** were able to reduce the cell viability of the MSSA and MRSA strains of *S. aureus*, showing that APDT is effective regardless of the AMR presented by the bacterium. The highest inactivation of both MSSA and MRSA was obtained using free Pc **4nInOAc** as the PS, with a reduction of 2.57 and 2.28 logs, respectively. The reduction of the photodegradation rate caused by the PS aggregation favored the action of free **4nInOAc**. However, the encapsulation of the PS decreased the photodynamic efficiency in the inactivation of bacteria because the negative charges present on the PHB nanoparticle surface as well in the *S. aureus* membrane hamper the interaction between the nanoparticle and the bacterial cell membrane. The antimicrobial action of the free and encapsulated **3nInOAc** and **4nInOAc** was considered moderated since the viability was reduced by an average of 2 logs (99%) of bacterial colonies of MSSA and MRSA strains.

## Abbreviations

AMR: antimicrobial resistance
APDT: antimicrobial photodynamic therapy
ANOVA: analysis of variance
ATCC: American type culture collection
BSA: bovine serum albumin
CFU: colony forming unit
DF: degrees of freedom
DMSO: dimethyl sulfoxide
EE: entrapment efficiency
ER: efficacy of nanoparticle recovery
FDA: Food and Drug Administration
GC: growth control
IAAS: integrate area of absorbance spectra
LC: light control
MMFF94s: variant of the Merck molecular force field
MRSA: methicillin-resistant *Staphylococcus aureus*
MS: mean squares
MSSA: methicillin-susceptible *Staphylococcus aureus*
PBS: phosphate-buffered saline
PHB: poly(3,hydroxybutyrate)
PM7: parametric method number 7
PS: photosensitizer
PVA: poly(vinyl alcohol)
ROS: reactive oxygen species
*S. aureus*: *Staphylococcus aureus*
SEM: scanning electron microscopy
SS: sum of squares
Trp: tryptophan
TSA: trypticase soy agar
TSB: trypticase soy broth
UFF: universal force field
WHO: World Health Organization
**3nInOAc**: 2(3),9(10),16(17),23(24)-Tetrakis[7-oxo-3-(3′,4′,5′-trimethoxyphenyl) coumarin] phthalocyaninato indium(III) acetate
**4nInOAc**: 1(4),8(11),15(18),22(25)-Tetrakis[7-oxo-3-(3′,4′,5′-trimethoxyphenyl) coumarin] phthalocyaninato indium(III) acetate
